# Subtype-specific collaborative transcription factor networks are promoted by OCT4 in the progression of prostate cancer

**DOI:** 10.1038/s41467-021-23974-4

**Published:** 2021-06-18

**Authors:** Ken-ichi Takayama, Takeo Kosaka, Takashi Suzuki, Hiroshi Hongo, Mototsugu Oya, Tetsuya Fujimura, Yutaka Suzuki, Satoshi Inoue

**Affiliations:** 1 Department of Systems Aging Science and Medicine, Tokyo Metropolitan Institute of Gerontology, Tokyo, Japan; 2 Department of Urology, Keio University School of Medicine, Tokyo, Japan; 3 Department of Pathology and Histotechnology, Tohoku University Graduate School of Medicine, Sendai, Miyagi Japan; 4 Department of Urology, Jichi Medical University, Shimotsuke, Tochigi Japan; 5 Department of Medical Genome Sciences, Graduate School of Frontier Sciences, The University of Tokyo, Chiba, Japan; 6 Division of Gene Regulation and Signal Transduction, Research Center for Genomic Medicine, Saitama Medical University, Saitama, Japan

**Keywords:** Prostate cancer, Nuclear receptors, Transcription

## Abstract

Interactive networks of transcription factors (TFs) have critical roles in epigenetic and gene regulation for cancer progression. It is required to clarify underlying mechanisms for transcriptional activation through concerted efforts of TFs. Here, we show the essential role of disease phase-specific TF collaboration changes in advanced prostate cancer (PC). Investigation of the transcriptome in castration-resistant PC (CRPC) revealed OCT4 as a key TF in the disease pathology. OCT4 confers epigenetic changes by promoting complex formation with FOXA1 and androgen receptor (AR), the central signals for the progression to CRPC. Meanwhile, OCT4 facilitates a distinctive complex formation with nuclear respiratory factor 1 (NRF1) to gain chemo-resistance in the absence of AR. Mechanistically, we reveal that OCT4 increases large droplet formations with AR/FOXA1 as well as NRF1 in vitro. Disruption of TF collaborations using a nucleoside analogue, ribavirin, inhibited treatment-resistant PC tumor growth. Thus, our findings highlight the formation of TF collaborations as a potent therapeutic target in advanced cancer.

## Abstract (web summary)

Transcription factors (TFs) often form distinct networks to regulate transcriptional program during cancer progression. Here the authors show that OCT4 is a common transcriptional factor in two types of advanced PC and as such, OCT4 accelerates a TF complex formation with the FOXA1/AR in castration-resistant PC and NRF1 in neuroendocrine PC.

## Introduction

Prostate cancer (PC) is the most frequent tumor in men worldwide^1,2^. Although androgen withdrawal, anti-androgen therapy, and taxane chemotherapy may be effective for PC, many of these patients develop lethal therapy-resistant PC called castration-resistant PC (CRPC)^3^ or more aggressive PC with neuroendocrine features (NEPC)^4^. Amplification of androgen receptor (AR) signaling is key in the transition to CRPC^1–3,5^. Transcription factors (TFs) form collaboration networks for transcriptional activation to exhibit the cellular phenotype in pluripotent or cancer cells^6–10^. In PC cells, pioneer factor FOXA1 opens and loosens the chromatin by histone modification to recruit its associated TFs such as AR^6,9,11,12^. It is of note that distinct types of AR collaborations are important for activating AR pathways in CRPC^7–9^. In contrast, NEPC is characterized by low expression of AR and could be developed from AR-positive tumor via lineage plasticity induced by increased expression of other TFs, such as N-MYC^13^, SOX2^14^, and ONECUT2^15,16^. Thus, the high lethality of PC indicates the urgent need to identify molecular mechanisms or effectors to elucidate the regulation of gene expression by TF networks.

The physical process of liquid-liquid phase separation (LLPS) contributes to the assembly of several membrane-less organelles in mammalian cells^17–20^. The main feature is the spontaneous separation of a mixed solution into two phases of different concentration. Intrinsically disordered regions (IDRs) of proteins support LLPS development by forming phase-separated condensates^21,22^. Key TFs also undergo phase separation in vitro and condensate formation in vivo on super enhancers (SEs) in cells^18,23^. The activation domains of TFs bind to the mediator complex in multiple conformations, leading to fuzzy protein–protein interactions working for transcriptional activation^18,24,25^.

Here, we study the TF collaborations underlying transcriptional activation, which contribute to PC aggressiveness. This study demonstrates OCT4 as a key component of the TF complex in CRPC by collaborating with the FOXA1/AR in AR-positive and NRF1 in AR-negative PC, respectively. OCT4 is known as a master regulator for the pluripotent state of stem cells^26,27^ forming a core regulatory circuitry with SOX2/KLF4 to promote pluripotent cell fate^10^. Interestingly, in advanced PC cells, OCT4 is recruited to specific genomic loci to activate other TFs possibly through phase-separation on enhancers and promoters. Moreover, we reveal that disruption of OCT4-TF complex by a nucleoside analog, ribavirin, will be an effective strategy to inhibit the signals responsible for CRPC tumor growth. Our findings highlight the feasibility of targeting LLPS-mediated TF collaborations on regulatory regions as a potential strategy to reprogram treatment-resistant tumor cells for preventing further progression.

## Results

### OCT4 functions to activate the AR/FOXA1 axis by enhancing complex formation

**Fig. 1:**
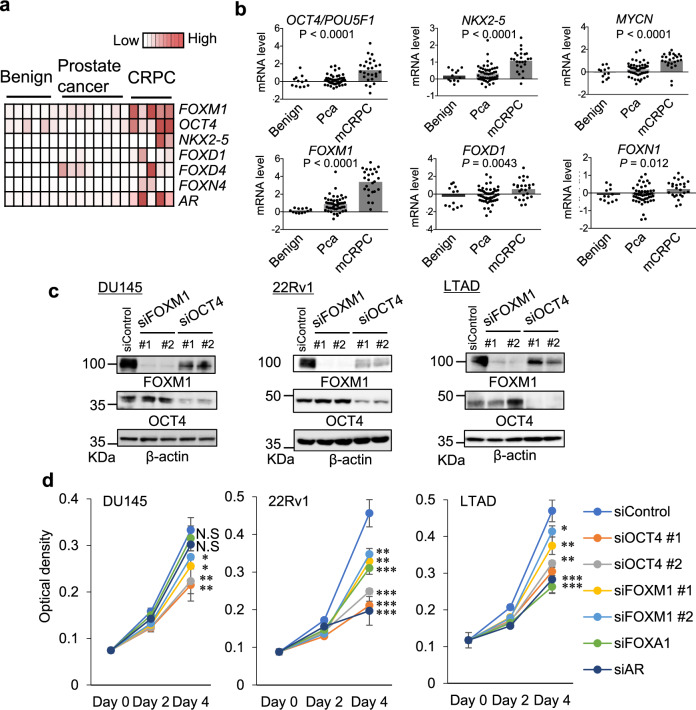
Identification of distinct TF networks in advanced PC that contributes to the aggressiveness. **a** Heatmap of TFs in benign prostate, primary PC, and CRPC tissues. Average RNA-seq results (RPKM) are summarized. Benign *N* = 6, Prostate cancer *N* = 8, CRPC *N* = 5. **b** Publicly microarray data (GSE35988) used for gene expression analysis. Pca: prostate cancer, mCRPC: metastatic CRPC. Two-sided Mann–Whitney test was performed between Pca and mCRPC. Benign *N* = 12, Pca *N* = 51, mCRPC *N* = 25). **c** Knockdown of FOXM1 and OCT4 expression by siRNAs. **d** Cell proliferation assay of multiple PC cells transfected with siControl or two siRNAs targeting indicated TFs. MTS assay was used to quantify the cell growth rate (*N* = 4, biological independent). N.S not significant, *P < 0.05, **P < 0.01, ***P < 0.001. two-sided *t*-test was performed. Data are presented as average ± S.D. Source data are provided as a Source Data file.

To identify TF networks responsible for the aggressiveness of PC, we investigated gene expression profiles of transcription factors in our RNA-sequencing (RNA-seq) dataset containing CRPC samples^28^. The expression of a specific subset of TFs is upregulated in CRPC compared with localized PC and benign regions (Fig. 1a). We validated that OCT4, as well as FOXM1^29^, are robustly induced during the disease progression (Fig. 1a, b). Using two independent small interfering RNAs (siRNAs), we observed that OCT4 knockdown affects cell proliferation (Fig. 1d) as well as FOXM1 protein levels (Fig. 1c) in several CRPC model cells (DU145, 22Rv1 and long-term androgen deprivation (LTAD) cells derived from hormone therapy-sensitive LNCaP cells). We previously observed that the high OCT4-expressing PC cells exhibited increased tumorigenicity and resistance to chemotherapy^30^. It is also notable that the Discovery (DISC) cohort listed OCT4 as one of highly expressed TFs in metastatic CRPC tissues^15^. Thus, we assume that OCT4 could be another driver molecule. However, the specific OCT4-mediated signals contributing to the aggressiveness of the diseases are required to be investigated.

**Fig. 2:**
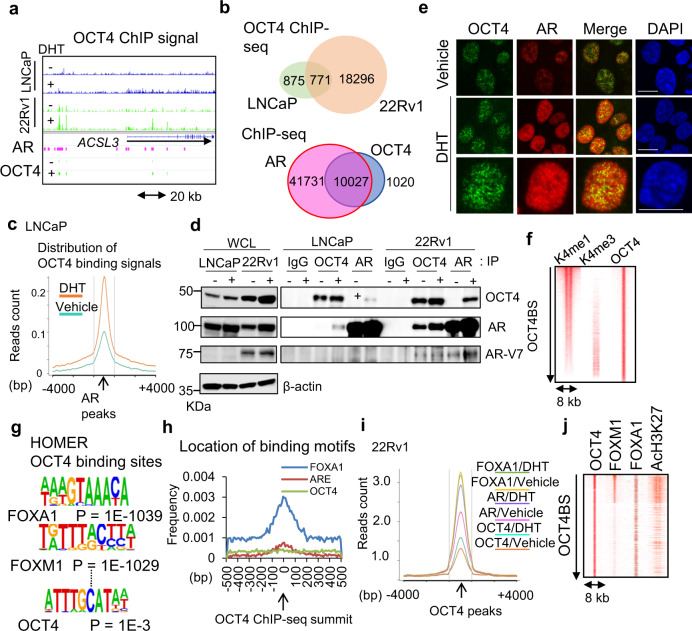
OCT4 functions as a modulator of AR and FOXA1 TF complex in AR-positive PC cells. **a** OCT4 ChIP-seq view of a representative AR-target gene, *ACSL3*. **b** (upper) OCT4-binding sites (OCT4BSs) by ChIP-seq in LNCaP and 22Rv1 cells. (lower) Venn diagram showing the overlap of AR with OCT4BSs in the presence of dihydrotestosterone (DHT) in 22Rv1 cells. **c** Composition plot of OCT4-binding signal in LNCaP cells. **d** Immunoblots of AR, AR-V7, and OCT4 after immunoprecipitation (IP) in LNCaP and 22Rv1 cells. **e** Immunofluorescence (IF) images of AR and OCT4 in 22Rv1 cells. Bar =  10 μm. **f** Heatmap of ChIP-seq signals showing the aligned peaks of active promoter (K4me3) and enhancer (K4me1) markers at OCT4 center. **g** Motif discovery analysis of OCT4 peak positions (±50 bp). HOMER, publicly available program, was used with the default settings to find the enriched motifs and *P*-value. **h** Distribution of FOXA1/AR/OCT motifs around OCT4-binding peaks. **i** Composition plots of FOXA1 and AR-binding signals around the peaks of OCT4-binding in 22Rv1 cells. **j** Heatmap of aligned ChIP-seq signals of FOXA1, FOXM1, and AcH3K27 at OCT4 center. Source data are provided as a Source Data file.

Therefore, we focused on dissecting the role of OCT4 in advanced stages of PC. To analyze the genomic action of OCT4 in PC cells, we performed chromatin immunoprecipitation combined with sequencing (ChIP-seq) study for global mapping of OCT4-binding sites (OCT4BSs) in LNCaP cells, and CRPC model 22Rv1 cells (Fig. 2a and Supplementary Fig. 1a, b). Strikingly, the number of obtained OCT4BSs in 22Rv1 cells was larger than that of LNCaP in line with the high protein level of OCT4 in 22Rv1 cells (Fig. 2b and Supplementary Fig. 1c). Majority of OCT4BSs overlapped with AR-binding sites (ARBSs) in 22Rv1 cells (Fig. 2b). Around the signal peaks of significant AR-binding sites (ARBSs), ChIP-seq signals of OCT4 increased in the presence of dihydrotestosterone (DHT) in androgen-sensitive LNCaP cells (Fig. 2c). The interaction of OCT4 with AR in the presence of DHT was observed (Fig. 2d). Immunofluorescence (IF) analysis revealed the co-localization of AR with OCT4 in the nucleus (Fig. 2e and Supplementary Fig. 1d). Moreover, overlap of OCT4 with active histone marks in 22Rv1 cells suggest the association of OCT4 with opened chromatin to activate gene expression (Fig. 2f).

Interestingly, HOMER analysis^31^ revealed that forkhead family motifs were highly enriched in the OCT4 ChIP-seq peak positions (Fig. 2g, h). In contrast, OCT4 in embryonic stem cells functions through OCT4/SOX2/NANOG motifs^10^. This finding motivated us to explore the unique role of OCT4 in the TF complex on enhancers in CRPC cells. We first investigated whether OCT4 association with enhancer regions are dependent on FOXA1. In ChIP-seq data, we observed that FOXA1 bindings are more enriched around the center of OCT4BSs than another FOX family TF, FOXM1 (Fig. 2i, j). We showed that binding of OCT4 to FOXA1 is robustly enhanced by AR addition (Supplementary Fig. 1e). Direct interaction was also analyzed by purified OCT4, FOXA1, and AR proteins (Supplementary Fig. 1f). In gel shift assays, we observed genomic binding of OCT4 is dependent on FOXA1-motif and complex formation with FOXA1/AR (Supplementary Fig. 1g). These results indicate that the recruitment of OCT4 to the enhancer regions stimulates and is dependent on AR/FOXA1 binding.

**Fig. 3:**
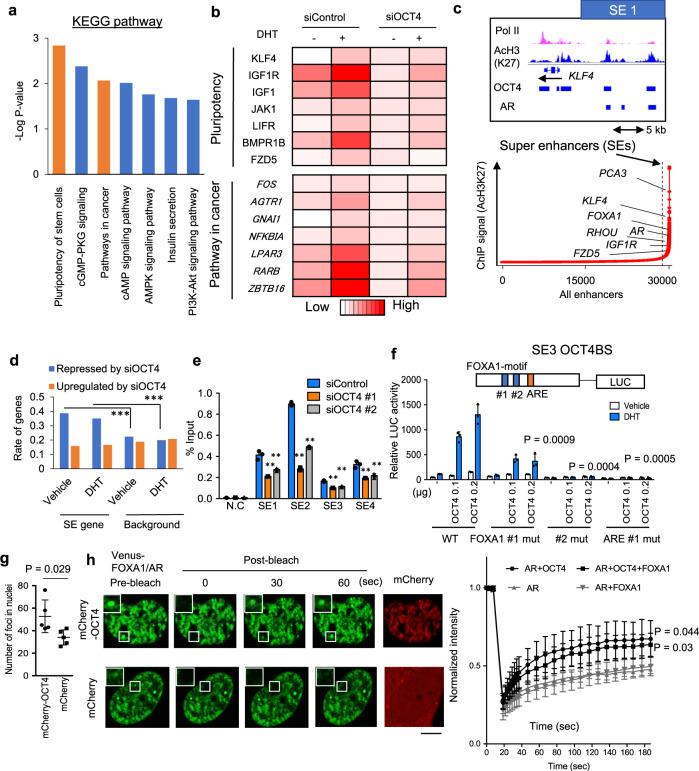
The role of OCT4 in superenhancer (SE) establishments to facilitate AR transcription program. **a** KEGG-pathway analysis of AR/OCT4 target genes. AR- and OCT4-binding genes were closest genes to binding sites. We selected androgen-induced genes (>1.5 fold) repressed by siOCT4 #1 (fold < 0.7) by microarray. **b** Heatmap showing representative genes regulated by OCT4 and AR. **c** Identification of Superenhancers (SEs) in advanced PC cell model. ROSE analysis of AcH3K27 ChIP-seq signals in 22Rv1 cells. Representative genes in the vicinity of SEs are indicated. Signal tracks of SE signal (AcH3K27), RNA pol II reads on four representative SE genes. **d** Repressed (Fold < 0.5) or induced (Fold > 1.5) genes by OCT4 silencing in microarray data. Significant enrichment of repressed genes among SE genes were determined by two-sided chi-square test. ***P < 0.0001. **e** ChIP assay of AcH3K27 was performed (*N* = 3, technical replicates). 22Rv1 cells were treated with siControl, siOCT4 #1, or #2 for 48 h. **P < 0.01. two-sided *t*-test was used to compare siOCT4 with siControl. Data are presented as average ± S.D. **f** (Upper) Luciferase vectors (wild type or mutation of FOXA1 motif #1, #2 or ARE) were constructed by inserting OCT4-binding sequence to pGL3-promoter vector. (Lower) LNCaP cells were transfected with luciferase vectors complexed with HA-OCT4 or control vector. Cells were then treated with DHT or vehicle for 24 h and luciferase assay was performed (*N* = 3, biological replicates). Two-sided *t*-test was performed to compare with WT. Data are presented as average ± S.D. **g** Venus-FOXA1 and Venus-AR were co-transfected with mCherry or mCherry-OCT4 into 293T cells. The number of nuclear foci is counted and quantified (*N* = 5, biological independent cells). Two-sided *t*-test was performed. Data are presented as average ± S.D. **h** FRAP kinetic plots and representative images from pre- and post-bleaching cells (*N* = 4, biological independent cells). 293T cells were transfected with (1) Venus-FOXA1, Venus-AR, and mCherry-OCT4 (AR + FOXA1 + OCT4), (2) Venus-AR and mCherry-OCT4 (AR + OCT4), (3) Venus-FOXA1, Venus-AR and mCherry (AR + FOXA1), or (4) Venus-AR and mCherry (AR). Representative images of (1) and (3) are shown. Cells were treated with DHT for 2 h. Bar = 10 μm. Two-sided *t*-test was performed. Data are presented as average ± S.D. Source data are provided as a Source Data file.

Next, we focused on the role of OCT4 in AR transcriptional program in CRPC. The gene expression profiles with OCT4 depletion were globally analyzed in CRPC model 22Rv1 cells using microarray. Notably, most of androgen-regulated genes were repressed by OCT4 knockdown, suggesting the importance of OCT4 in androgen signaling (Supplementary Fig. 2a). We found that androgen-regulated genes with AR- and OCT4-bindings were associated with signal transductions in cells, particularly pluripotency of stem cells and pathways in cancer (Fig. 3a, b). We further explored whether OCT4 bindings contribute to the super enhancer (SE) formation^24,32,33^ in CRPC cells. We analyzed AcH3K27 ChIP-seq result with Ranking of Super Enhancer (ROSE) program to determine specific SE regions in 22Rv1 cells (Fig. 3c). Interestingly, OCT4 occupancy was observed at high-ranking SEs (Fig. 3c and Supplementary Fig. 2b). In addition, SE genes, which are closest to SE regions, are significantly repressed by OCT4 knockdown using microarray data (Fig. 3d). In line with the result, ChIP analysis showed reduction of AcH3K27 level as well as AR bindings at SE regions by OCT4 silencing (Fig. 3e and Supplementary Fig. 2c). Thus, these results suggest that OCT4 contributes to the SE activity to enhance the transcription of important oncogenes regulated by AR in CRPC cells.

Furthermore, we analyzed the functional property of OCT4 binding by luciferase assay. OCT4-dependent activation of AR activity (Supplementary Fig. 2d) was diminished by mutations of FOXA1/AR binding sequence in luciferase assay (Fig. 3f). We also showed the importance of both IDRs at C-terminal and N-terminal of OCT4 in the association with AR and AR-mediated gene induction and cell growth (Supplementary Fig. 3a–c). To examine the physicochemical feature of OCT4 binding on AR enhancers, we next evaluated AR dynamics using fluorescence recovery after photobleaching (FRAP). 293T cells transfected with Venus-AR/FOXA1 were treated with DHT. We observed that OCT4-mCherry addition significantly increased the number of AR/FOXA1 foci in nuclei (Fig. 3g) and enhanced AR turnover with rapid recovery, suggesting OCT4 action to promote AR mobility in the nucleus (Fig. 3h). This is consistent with the results of luciferase assay showing enhanced AR transcriptional activity by OCT4 addition in 293T cells (Supplementary Fig. 4a). Similarly, OCT4 overexpression activated the recovery in LNCaP cells, although OCT4 lacking IDR regions failed (Supplementary Fig. 4b). In contrast, OCT4 knockdown repressed AR turnover in 22Rv1 cells (Supplementary Fig. 4c, d) as well as AR transcriptional activity in luciferase assay (Supplementary Fig. 4e). Taken together, these data imply that OCT4 has a unique impact on AR signaling by regulating physical aspect of AR and FOXA1 genomic action through IDRs.

### Disruption of OCT4-dependent induction of AR pathway by drug treatments

**Fig. 4:**
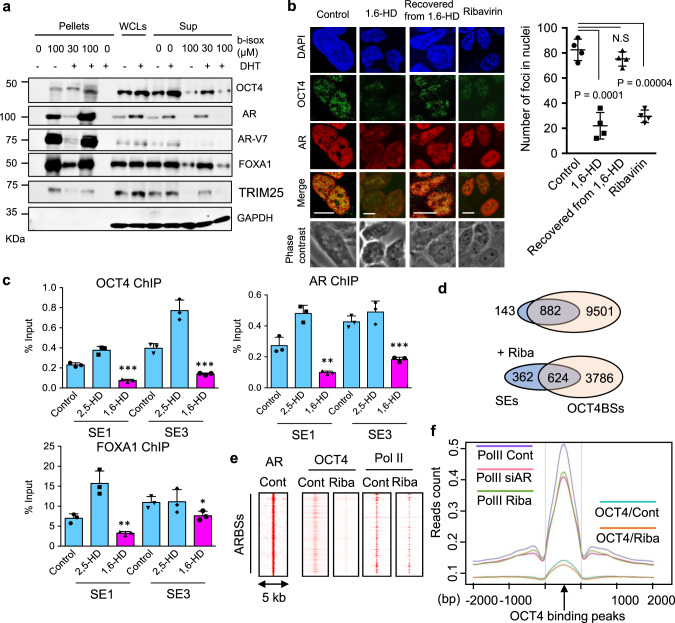
The effect of 1,6-HD and a nucleoside analog ribavirin (Riba) on OCT4-occupied enhancer activity. **a** Immunoblots showing that AR, FOXA1, and OCT4 are precipitated by biotinylated isoxazole (b-isox). TRIM25, a representative RNA-binding protein, was used as a positive control. GAPDH was used as a negative control. WCLs: whole cell lysates, Sup: supernatants. **b** (Left) IF images of AR and OCT4 in 22Rv1 cells. Cells were treated with 1,6-hexandiol (HD) for 5 min, recovered for 1 h after 1,6-HD for 5 min, or ribavirin for 24 h. Bar = 10 μm. (Right) Quantification of OCT4 puncta (*N* = 4, biological independent cells) Two-sided *t*-test was performed. N.S not significant. Data are presented as average ± S.D. **c** ChIP analysis was performed in 22Rv1 cells treated with DHT for 24 h (*N* = 3, technical replicates). Cells were treated with 2,5-HD, 1,6-HD, or vehicle for 5 min before fixation. *P < 0.05, **P < 0.01, ***P < 0.001 vs Control. Two-sided *t*-test was performed. Data are presented as average ± S.D. **d** Venn diagram showing the overlap of SE regions with OCT4BSs in the absence or presence of ribavirin. **e** Heatmap of ChIP-seq signals showing the aligned peaks of RNA pol II and OCT4-binding signals at AR binding sites (ARBSs). Cont: control. **f** Composition plot of RNA pol II and OCT4-binding signal around the OCT4-binding peaks. Cells were treated with siAR or ribavirin. Source data are provided as a Source Data file.

OCT4 has two intrinsically disordered activation domains (ADs) responsible for gene activation^18,30^. Stretches of amino acids in ADs form IDRs that contribute to phase separation. We revealed that components containing OCT4 as well as AR/FOXA1 were precipitated from lysates in the presence of biotinylated isoxazole (b-isox), a compound that precipitates proteins with IDR enriched in phase-separated granules^23,34,35^ (Fig. 4a). Because we observed granular dots, OCT4 puncta, in the nucleus by IF analysis (Fig. 2e), we next examined whether such condensates have a property of phase separation. 1,6-hexandiol (1,6-HD) is an aliphatic alcohol that disrupts weak hydrophobic interactions involved in phase-separated ribonuleoprotein granules and membrane-less structures^36,37^. Using 1,6-HD, we observed that the extent of nuclear condensates of OCT4 was reduced and AR exported out to the cytoplasm (Fig. 4b), reducing the recruitment of AR/OCT4 complex to enhancers although another aliphatic alcohol (2,5-HD) with minimal impact on IDR proteins^23^ did not affect the recruitment significantly (Fig. 4c). The disruption of the AR complex was recovered by eliminating 1,6-HD treatment, indicating the fluid-like characteristic. Taken together, these results would indicate that hydrophobic interaction plays a role in the assembly of AR/OCT4 complex on genome.

We previously identified a nucleoside analog, ribavirin, as a conversion modulator from drug resistance to sensitivity in OCT4-high expressing PC cells^30^. Ribavirin resembles purine RNA nucleotides and prevents the proliferation of RNA viruses^38,39^. Although a few studies in cancer cells suggest some roles of ribavirin^40–42^, the mechanistic aspect of ribavirin in PC remains unknown. We therefore investigated the molecular mechanism of ribavirin action in CRPC cells. IF analysis revealed that the number and size of OCT4 puncta were reduced by ribavirin treatment (Fig. 4b) as observed in the treatment of 1,6-HD. We next analyzed the effect of ribavirin on OCT4 and AR-mediated transcriptional activation by ChIP-seq. While majority of SEs are widely overlapped with OCT4BSs, OCT4 ChIP-seq showed ribavirin treatment affected OCT4-ocupancy and reduced the overlap of SEs with OCT4BSs (Fig. 4d and Supplementary Fig. 5a). Interestingly, we found FOXM1, which is a target of OCT4, was robustly repressed by ribavirin (Supplementary Fig. 5b). Notably, RNA pol II-binding peaks repressed by ribavirin significantly overlapped with those inhibited by siAR, suggesting that ribavirin repressed AR-associated signaling (Supplementary Fig. 5c). Consistently, around AR-binding peaks, we observed that binding of both OCT4 and RNA pol II were suppressed (Fig. 4e). In OCT4-binding peaks, enhancer/transcriptional activity represented by RNA pol II occupancy was diminished by ribavirin treatment as observed by AR knockdown (Fig. 4f). RNA-seq data showed that androgen signals were inhibited by ribavirin (Supplementary Fig. 5d). Inhibition of AR complex formation with OCT4 and FOXA1 was observed by western blot analysis (Supplementary Fig. 5e, f). Thus, these results demonstrated that ribavirin inhibits the formation of OCT4-AR axis by modulating OCT4 condensates in the nucleus.

### NRF1 binds to OCT4 to promote chemoresistance in AR-negative PC

**Fig. 5:**
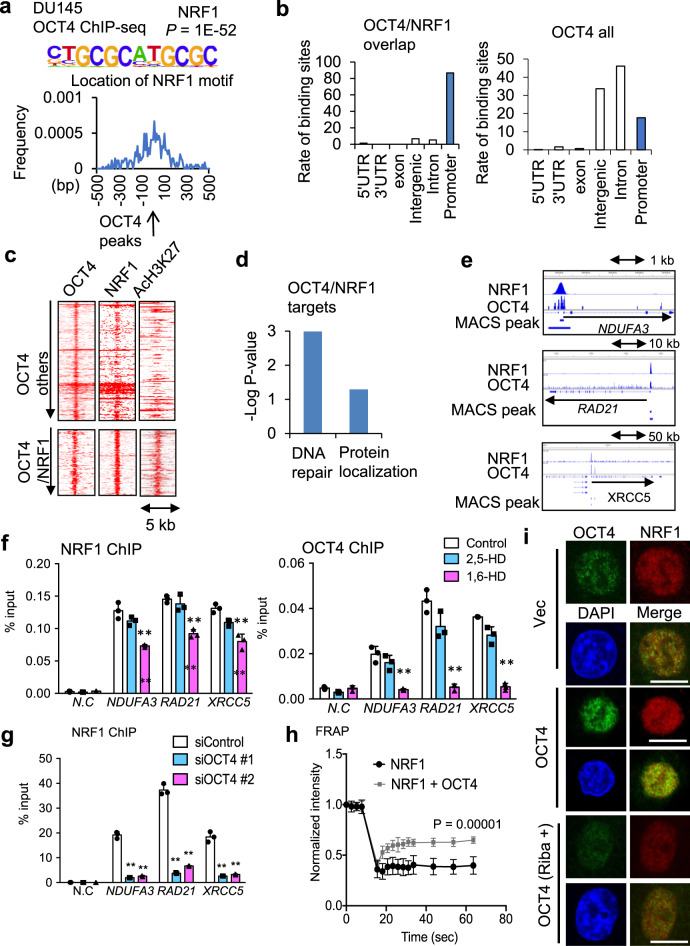
Distinct TF complex formation of OCT4 with NRF1 in AR-negative PC cells. **a** HOMER analysis of OCT4-binding peaks in DU145 cells. **b** Distribution of OCT4BSs in the genomic regions around the closest gene was analyzed. **c** Heatmap of ChIP-seq signals shows the aligned peaks of NRF1, AcH3K27 signals at OCT4-binding peaks of control cells. **d** Microarray analysis was performed to analyze gene expression. Significantly enriched gene ontologies (GOs) among OCT4 -regulated genes with OCT4/NRF1 bindings (Fold < 0.5 by siOCT4 #1) in DU145 cells are summarized. **e** ChIP-seq views of NRF1 and OCT4 reads on three representative OCT4-regulated genes. Significant OCT4 and NRF1-binding regions (P < 1.0E-4) are shown by boxes. **f** The effect of 1,6-HD (hexandiol) on chromatin recruitments of NRF1 and OCT4. ChIP analysis was performed in DU145 cells (N = 3, technical replicates). Cells were treated with 2,5-HD, 1,6-HD or vehicle for 5 minutes before fixation. N.C: negative control. **P < 0.01. Two-sided *t*-test was performed. Data are presented as average ± S.D. **g** DU145 cells were treated with siControl, siOCT4 #1, or #2 for 48 h. ChIP analysis was performed using anti-NRF1 and anti-OCT4 antibodies (*N* = 3, technical replicates). **P < 0.01. Two-sided *t*-test was performed. Data are presented as average ± S.D. **h** FRAP kinetic plots from pre- and post-bleaching cells (NRF1:*N* = 5, NRF1 + OCT4:*N* = 6, biological independent cells). 293T cells were transfected with Venus-NRF1 or mCherry-OCT4. Two-sided *t*-test was performed. Data are presented as average ± S.D. **i** IF images of NRF1 and OCT4 in DU145 cells overexpressing OCT4 or control cells. OCT4-overexpressing cells were treated with vehicle or ribavirin for 48 h. Bar = 10 μm. Source data are provided as a Source Data file.

NEPC, that is characterized by low expression of AR, could be developed from AR-positive tumor by lineage plasticity induced by transcriptional programs^13–16^. We next explored OCT4 signaling in AR-negative PC DU145 cells to investigate the AR-independent role of OCT4. Global OCT4BSs in the AR-negative DU145 cells were determined by OCT4 ChIP-seq analyses. A total of 552 certain OCT4BSs was obtained, and HOMER analysis of binding peaks revealed that nuclear respiratory factor 1 (NRF1) binding motif was enriched at the center of OCT4BSs (Fig. 5a and Supplementary Fig. 6a). NRF1 is a regulator of mitochondrial oxidative phosphorylation via transcriptional activation of these components in neurons and adipose tissues^43–45^. Our NRF1 ChIP-seq study demonstrated that NRF1 binds to the genome through the NRF1-binding motif (Supplementary Fig. 6b). Global NRF1 associations with genome were detected at OCT4BSs, however NRF2^46^ ChIP-seq analysis showed no significant overlap (Supplementary Fig. 6c), suggesting that this association is specific to NRF1. Overlapped regions of NRF1-binding sites with OCT4BSs were concentrated at the promoter regions (Fig. 5b). AcH3K27 acetylation was strongly enhanced around these overlapped regions (Fig. 5c). In addition, association of NRF1, but not NRF2 with OCT4 was observed in immunoprecipitation and western blot assay (Supplementary Fig. 6d). To investigate the possibility that OCT4 undergoes transcriptional activation with NRF1 in DU145 cells, we precipitated enrichment of OCT4 and NRF1 protein complex in proteins with IDR using b-isox (Supplementary Fig. 6e). These findings suggest that OCT4 interacts with NRF1 to increase promoter activity.

Next, we further dissected the molecular events following OCT4 function in the aggressiveness of AR-negative PC by analyzing transcriptome profiling in DU145 cells. We selected OCT4-binding genes repressed by siOCT4 (Fold < 0.5), and identified functional OCT4 target gene signature. Gene ontology (GO) analysis revealed that OCT4/NRF1-regulated genes were associated with biological processes such as DNA repair related genes, including *XRCC5* and *RAD21* (Fig. 5d, e). It is supposed that alterations of the DNA repair signaling pathway confer chemoresistance in cancer cells^47,48^. 1,6-HD treatment reduced OCT4 and NRF1 recruitment in NRF1/OCT4-binding regions of target genes (Fig. 5f). We showed that NRF1 recruitment to the promoters of these target genes was inhibited by OCT4 silencing (Fig. 5g). Gel shift assay showed the association of NRF1 with OCT4 induced DNA binding at the NRF1 motif (Supplementary Fig. 6f, g). To examine the physicochemical feature of OCT4 on NRF1 function, we evaluated NRF1 dynamics by FRAP. We then observed that OCT4 addition significantly enhanced NRF1 turnover and rapid recovery within 10 s, suggesting that OCT4 enhances NRF1 mobility in the nucleus (Fig. 5h and Supplementary Fig. 6h, i). Moreover, we observed that ribavirin treatment inhibits the number of OCT4 puncta in the nucleus and reduced NRF1 expression (Fig. 5i). Consistently, ribavirin treatment caused a shift in OCT4/NRF1-binding regions (Supplementary Fig. 7a–c). These findings support the possibility that OCT4 facilitates NRF1 recruitment to the promoter for gene expression and that ribavirin alleviated the collaborative function.

**Fig. 6:**
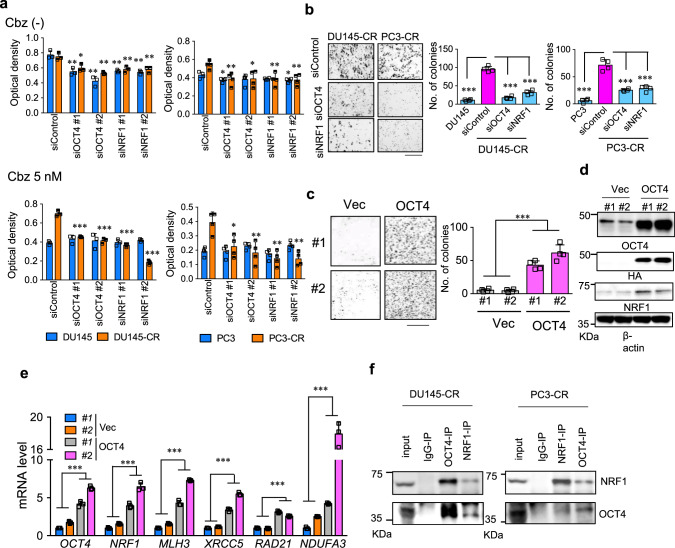
OCT4 cooperates with NRF1 to activate its target. **a** Growth inhibition of chemotherapy resistant PC cells by siRNA-mediated knockdown of OCT4 and NRF1. Chemoresistant PC cells (PC3-CR and DU145-CR) and their parental cells were treated with cabazitaxel (Cbz) 5 nM or vehicle. Cells were transfected with siControl, two siRNAs targeting OCT4 (#1, #2) or two siRNAs targeting NRF1(#1, #2). After 72 h incubation, cell viability was measured by MTS assay. *N* = 4, biological independent samples. Two-sided *t*-test was performed. *P < 0.05, **P < 0.01, ***P < 0.001. Data are presented as average ± S.D. **b** Representative colony formation assays and quantification of cells following 72 h treatment with cabazitaxel 10 nM. DU145-CR, PC3-CR, and parental cells were transfected with siControl, siOCT4 #1, or siNRF1 #1 (10 nM). *N* = 4, biological independent samples. Two-sided *t*-test was performed. ***P < 0.001. Data are presented as average ± S.D. Bar = 100 μm. **c** Representative colony formation assays and quantification of cells following 72 h treatment with cabazitaxel 10 nM. DU145 cells stably expressing HA-OCT4 (#1 and #2) or empty vector (Vec) (#1 and #2) were used. *N* = 4, biological independent samples. Two-way ANOVA was performed. ***P < 0.001. Data are presented as average ± S.D. Bar = 100 μm. **d** Immunoblotting of OCT4, HA, and NRF1 was performed in DU145 cells stably expressing HA-OCT4 (#1 and #2) or empty vector (Vec) (#1 and #2). **e** qRT-PCR analysis to measure mRNA levels of OCT4 target genes, OCT4 and NRF1 in DU145 cells stably expressing HA-OCT4 (#1 and #2) or empty vector (Vec) (#1 and #2). *N* = 3, technical replicates. Two-way ANOVA was performed. ***P < 0.001. Data are presented as average ± S.D. **f** Interaction of OCT4 with NRF1 in chemoresistnat PC cells. Immunoblots after immunoprecipitation (IP) by anti-NRF1 and OCT4 antibodies in DU145-CR and PC3-CR cells. Source data are provided as a Source Data file.

We further analyzed the role of OCT4/NRF1 in the advanced lethal PC cells using cell models derived from DU145 and PC3 cells, which mimic the high tumorigenicity and drug (cabazitaxel (Cbz))^49^ resistant phenotype (DU145-CR, PC3-CR cells^50^). Compared with parental cells, OCT4-binding genes were more significantly repressed by OCT4/NRF1 silencing (Supplementary Fig. 8a). We found that Cbz-resistance was almost diminished by OCT4 and NRF1 knockdown in both cell lines (Fig. 6a, b). Concurrently, OCT4 overexpression in parental DU145 cells showed increased number of colonies after Cbz treatment compared with control cells by inducing its target genes (Fig. 6c–e). Interaction between OCT4 and NRF1 was also confirmed in these Cbz-resistant PC cells (Fig. 6f). Interestingly, our analyses suggested that these two TFs regulate mutual expression by binding each other (Supplementary Fig. 8b). Indeed, knockdown of OCT4 inhibited NRF1 expression and vice versa in both parental and aggressive PC cells (Supplementary Fig. 8c). To illustrate the role of these TFs in the acquirement of chemoresistance, we analyzed the global change of gene expressions in both aggressive cell lines by decreasing the expression of OCT4 and NRF1. As expected, OCT4- and NRF1-regulated genes were significantly overlapped in microarray analysis (Supplementary Fig. 8d). We found that genes regulated by ribavirin treatment are significantly enriched among OCT4- and NRF1-regulated genes, suggesting that ribavirin modulates OCT4 signaling pathway in chemoresistant PC cells (Supplementary Fig. 8e). Thus, regulation of transcription by OCT4/NRF1 contributes to chemoresistant phenotype independent of AR.

### Droplet formation of collaborating TFs is enhanced by OCT4 in vitro

**Fig. 7:**
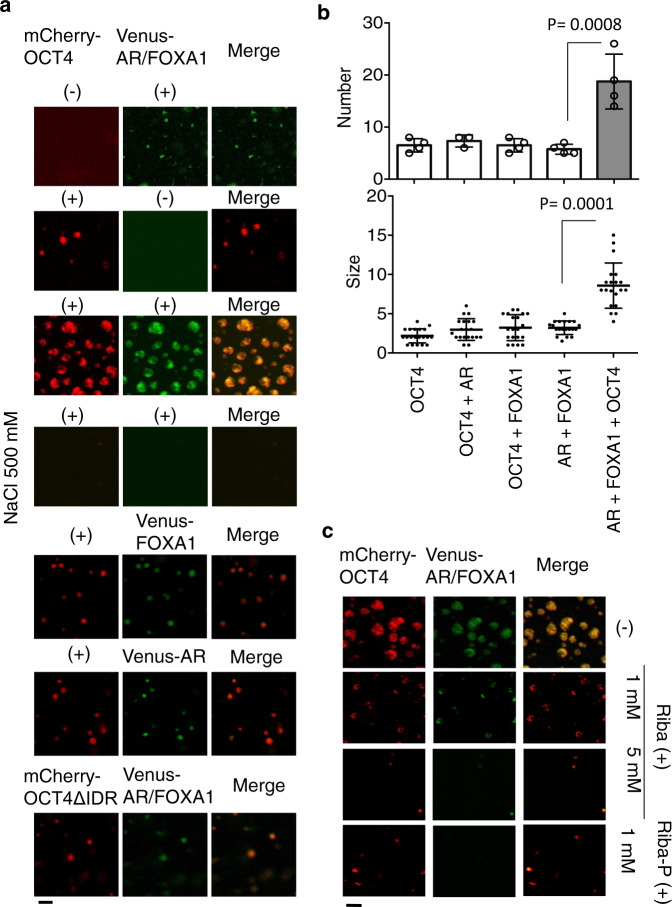
Enhanced liquid droplet formation by collaborating TF complexes in vitro. **a** Representative images of droplet formation of mCherry, Venus, mCherry-OCT4, Venus-FOXA1, and Venus-AR (total 10 μM) as indicated in droplet formation buffer with 125/500 mM NaCl and 10% PEG-8000. Bar = 10 μm. **b** Quantification of droplet formations of mCherry-OCT4, Venus-FOXA1, and Venus-AR in the presence of DHT. Average numbers (AR, AR + FOXA1 + OCT4: *N* = 4, others: *N* = 5) or sizes (*N* = 20) of droplets. One-way ANOVA and post-doc Dunnett’s test were performed. Data are presented as average ± S.D. **c** Effects of ribavirin and ribavirin-phosphate (P) on the droplet formation. Representative images of droplet formation of mCherry-OCT4, Venus-FOXA1, and Venus-AR (total 10 μM), as indicated in droplet formation buffer with 125 mM NaCl and 10% PEG-8000. Bar = 10 μm. Source data are provided as a Source Data file.

We next sought to determine whether TFs in the OCT4 complex are involved in formation of phase-separated droplets in vitro by droplet assay^18^. Purified Venus-AR, Venus-FOXA1, and mCherry-OCT4 proteins were then added, turning the solution opaque. In immunofluorescence analysis of the opaque mCherry-OCT4 and Venus-FOXA1/AR, we observed droplets freely moving in solution. The droplet formation assay showed that larger condensed droplets were formed by mixing mCherry-OCT4 and Venus-FOXA1/AR proteins compared with Venus-AR alone or Venus-FOXA1 alone with mCherry-OCT4, indicating the enhancement of droplet formation ability of AR complex by OCT4 (Fig. 7a, b). Interestingly, this change was dependent on DHT treatment (Supplementary Fig. 9a) and OCT4 concentration (Supplementary Fig. 9b). Droplet formations of Venus-FOXA1/AR and mCherry-OCT4 proteins was alleviated by increasing NaCl concentration (Fig. 7a), reflecting the biophysical properties of these droplets in phase separation. FRAP analysis revealed more rapid recovery of fluorescence in the presence of OCT4 (Supplementary Fig. 9c). To analyze the effect of ribavirin treatment on phase separation, we tested whether ribavirin affect the droplet formation. By IF assay, we observed that mCherry-OCT4 and Venus-FOXA1/AR droplets were reduced in size and number by the addition of ribavirin or phospho-ribavirin (Fig. 7c), suggesting that ribavirin act on the formed networks of weak protein–protein interactions. Similar results were obtained by mixture of mCherry-OCT4 and Venus-NRF1 (Supplementary Fig. 9d). By mixing both TFs, we observed large droplets and an increased number of droplets containing both proteins. This droplet formation was sensitive to high concentration of salt and ribavirin treatment (Supplementary Fig. 9e).

### Clinical significance of OCT4 and its collaborating factors in lethal PC tissues

**Fig. 8:**
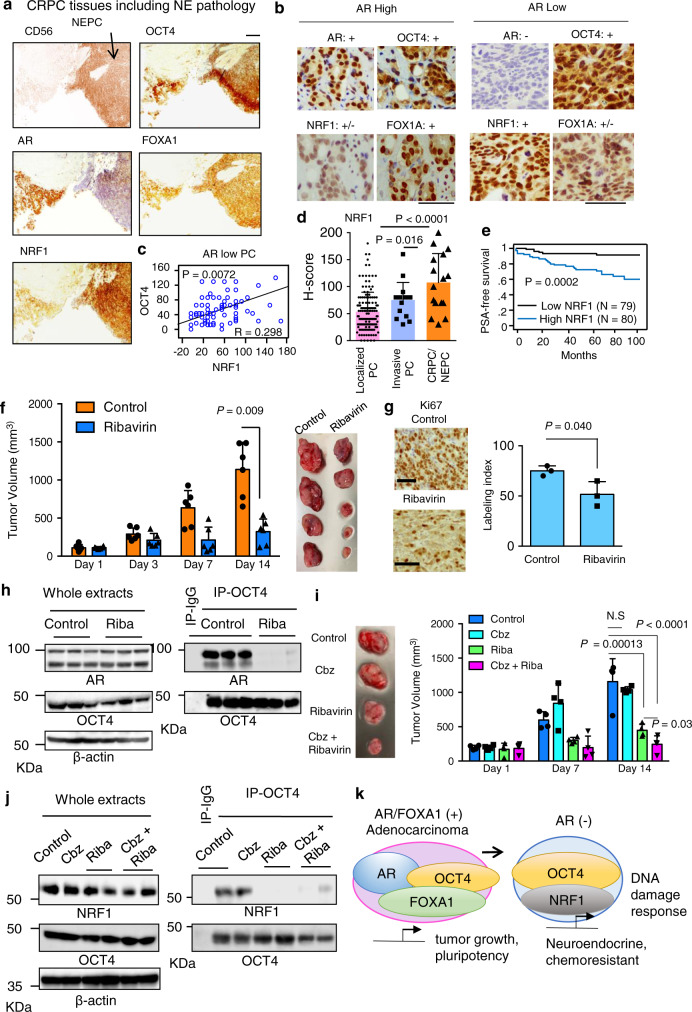
The OCT4-centered phase separation could be a promising therapeutic target of aggressive PC. **a** Immunohistochemistry (IHC) of OCT4, FOXA1, AR, CD56, and NRF1 in CRPC/NEPC tissues (*N* = 16). Bar = 100 μm. Representative images of NEPC pathology is shown. **b** Representative images of OCT4-associated TFs (in specimens with high or low expression of AR). +: strongly positive, +/−: weakly positive and −: negative. Bar = 50 μm. **c** The expression level of OCT4 protein detected by IHC correlated with NRF1 in AR low PC tissues (*N* = 80). Regression analysis was performed. **d** NRF1 protein level is upregulated in CRPC/NEPC tissues (*N* = 16) compared with localized PC tissues (*N* = 143) or locally invasive PC (*N* = 16). One-way ANOVA and followed Dunnett’s tests were performed. Data are presented as average ± S.D. **e** NRF1 is a prognostic factor for PC (*N* = 159). PC patients were classified into two groups according to the median value of NRF1 H-score. Survival analyses were performed using the Kaplan–Meir method and curves were compared by the log rank test. **f** Nude mice were inoculated with 22Rv1 cells. Mice were castrated after tumor development. Growth of tumors in nude mice treated with vehicle or ribavirin are shown (*N* = 6, biological independent animals). Two-sided *t*-test was performed. Data are presented as average ± S.D. **g** Immunohistochemical (IHC) images and labeling indexes of Ki67 in 22Rv1 tumor specimens (*N* = 3, biological independent samples). Two-sided *t*-test was performed. Data are presented as average ± S.D. Bar = 50 μm. **h** Immunoblots of AR and OCT4 after immunoprecipitation (IP) in 22Rv1 tumors. **i** Nude mice were inoculated with DU145-CR cells. (left) Mice were treated with vehicle, Cabazitaxel (Cbz), Ribavirin, Cbz +  Ribavirin. (right) Growth of tumors in nude mice treated with vehicle or ribavirin (Riba) are shown (*N* = 4, biological independent animals). One-way ANOVA and followed two-sided *t*-tests were performed. Data are presented as average ± S.D. **j** Immunoblots of NRF1 and OCT4 after immunoprecipitation (IP). **k** Schematic diagram of OCT4 function in PC progression. Source data are provided as a Source Data file.

OCT4 protein expression is markedly induced in CRPC tissues of patients resistant to chemotherapy^51^. This finding is consistent with the expression pattern in a public microarray or RNA-seq database (Fig. 1a, b). We then asked whether OCT4-associated enhancer complexes are involved in the aggressiveness of PC by immunohistochemical (IHC) analysis. By using CRPC/NEPC tissues obtained from patients with relapsed tumors, protein expression levels of OCT4 and other interacting TFs (AR, FOXA1, and NRF1) was evaluated (Fig. 8a, b). Some CRPC specimens focally included NEPC component (CD56: +, AR: −) (Fig. 8a). The protein expression level of OCT4 was higher in these CRPC/NEPC tissues compared with localized PC tissues (Supplementary Fig. 10a). Interestingly, OCT4 protein level is positively associated with AR in CRPC/NEPC tissues (Supplementary Fig. 10b). NRF1 expression was significantly associated with OCT4 expression in PC tissues with low AR expression (Fig. 8b, c and Supplementary Fig. 10c). Further analysis showed that NRF1 mRNA expression was significantly associated with OCT4 in NEPC enriched CRPC cohort and metastatic CRPC cohort, supporting the coordinated regulation at transcriptional level in CRPC/NEPC^52,53^ (Supplementary Fig. 10d, e). We found elevated NRF1 expression in CRPC/NEPC tissues compared with localized PC tissues (Fig. 8d and Supplementary Fig. 10f). Moreover, increased expression of both TFs is associated with high NEPC score^54^ (Supplementary Fig. 10g). Besides, high expression level of NRF1 in PC tissues correlated with poor prognosis of patients (Fig. 8e). Taken together, these results indicate that collaborating TF complex of OCT4 is associated with disease progression to the lethal PC.

### Targeting TF collaboration is effective for inhibiting treatment-resistant PC tumor growth

Epigenetic modification by TFs at SEs has a prominent role in diseased cellular states. We next aimed to test whether targeting TF collaborative networks could be a potent therapeutic target for cancer. In xenograft models of AR-positive 22Rv1 in castrated mice, marked inhibition of castration-resistant tumor growth was observed by ribavirin treatment (Fig. 8f). IHC analysis showed bionecrotic tissues (Supplementary Fig. 11a) and a decrease in the cell proliferation marker Ki67 by ribavirin treatment (Fig. 8g). Notably, tumor samples showed that interaction between OCT4 and AR was dramatically decreased (Fig. 8h), although ribavirin treatment decreased OCT4 protein levels in the nucleus of tumor cells (Supplementary Fig. 11a). This result suggests the repression of OCT4-centered TF complex formation in tumors. In xenograft model of DU145-CR tumors, we treated mice with vehicle, Cbz, ribavirin, or Cbz+ ribavirin. We observed that tumor growth was inhibited by ribavirin treatment (Fig. 8i). Combinational treatment by Cbz and ribavirin showed recovery of cabazitaxel effect. Notably, combinational treatment by Cbz and ribavirin showed recovery of cabazitaxel effect. Moreover, ribavirin treatment inhibits interaction between OCT4 with NRF1 in tumors (Fig. 8j) These results suggest the repression of OCT4-centered TF collaborations in tumors without obvious toxic effect (Supplementary Fig. 11b, c). To further analyze the efficacy of ribavirin treatment to cancer stem-like cell (CSC) maintenance of lethal PC, we tested the efficacy of ribavirin in patient-derived cell spheroid culture system^55,56^, which forms a sphere from clinical samples that has a multidrug resistant phenotype. Spheroid formation was inhibited by Cbz+ ribavirin, suggesting the efficacy of combining ribavirin with chemotherapy also in this patient-derived model (Supplementary Fig. 11d, e). Collectively, these results suggested growth inhibitory effects to alleviate the aggressiveness of CRPC/NEPC tumors and CSC maintenance upon combination therapy of ribavirin and chemotherapy (Fig. 8k).

## Discussion

We here demonstrated that TF network formation was dependent on cell circumstances with different expression patterns of TFs. We assume that collaborative TFs facilitate weak multivalent interactions through IDR regions to modulate the property of phase separation on enhancers/promoters, thus condensation of such collaborating factors creates a network. The interdependence affects TF activity to support a dynamic, disease-phase dependent model for diverse signals. It is likely that induced expression of these TF components during cancer progression enhances specific target gene expression. This study identified OCT4 as a key component of the TF complex in CRPC and NEPC tissues by collaborating with the FOXA1/AR in AR-positive and NRF1 in AR-negative PC, respectively. OCT4 consists of two stretched ADs with IDRs to condensate at SEs^18,21^. We found that OCT4 has a unique property in PC cells, as it is recruited to specific genomic loci to activate other TFs, such as AR/FOXA1 and NRF1. We revealed that OCT4 and NRF1 coordinately regulate DNA damage response signals such as DNA repair, which is involved in PC progression^47,48^. Notably, we found that NRF1 is highly expressed in concordance with OCT4 in CRPC/NEPC tissues in which AR is expressed at low levels. Thus, NRF1/OCT4 complex would be another candidate for transcriptional mechanisms involved in fatal NEPC development to facilitate cell viability in response to chemotherapy. Consistent with this model, NRF1 expression is significantly associated with poor clinical outcomes in PC patients, suggesting the role of NRF1 in aggressive PC. Furthermore, in DISC cohort, OCT4 was expressed most concordantly with ONECUT2, a driver gene for NEPC development^15^. Thus, it is possible that OCT4 functions through association with key TFs such as NRF1 and ONECUT2 to induce transcriptional program for PC progression.

The limited options for treating patients with CRPC/NEPC have emphasized on the need for identifying druggable targets^4^. Tumor cells have large SEs at driver oncogenes that are not found in cells that tumors are derived from, and are sensitive to drugs targeting SE components^57,58^. We assumed that OCT4 induced CRPC-associated SEs or promoters by promoting formation of TF collaborations possibly through phase separation. In addition, the present study suggested that the number of OCT4 puncta and in vitro droplets could be reduced with one of the nucleoside analogs, i.e. ribavirin, suggesting the disruption of OCT4-centered TF interactions. We speculate that the function as a nucleoside analog mimicking a ribonucleotide may affect OCT4 condensation, as RNA interaction with IDR protein can modify LLPS distribution^19,24,37^. Consistently, treatment with the 1, 6-HD, which is supposed to disrupt weak hydrophobic interactions observed in phase-separated granules^23^, diminished OCT4 condensates and AR or NRF1 nuclear action. In addition, we also found that OCT4 protein expression level in tumors was reduced by ribavirin. It is possible that ribavirin also affects the transcription or protein stability of OCT4 to reduce the OCT4 condensates on SEs, although this hypothesis should be tested in future studies. Targeting OCT4-mediated TF complex formation by ribavirin also suppressed tumor growth. Thus, we propose that repression of TF collaborations involved in phase separation on SEs or promoters could be a promising strategy for advanced cancer therapy. It would reprogram tumor cells towards increased sensitivity to chemotherapy and ultimately to attenuate cancer progression.

## Methods

### Cell culture

The cell lines used in the present study were obtained from American Type Culture Collection (ATCC). Identities of the cells were confirmed by short tandem repeat (STR) analyses (BEX co. Ltd., Tokyo, Japan). All cell lines were grown at 37 °C in a 5% CO_2_ atmosphere. VCaP and 293T cells were cultured in DMEM medium supplemented with 10% fetal bovine serum (FBS), 50 U/mL penicillin, and 50 μg/mL streptomycin. DU145, PC3, 22Rv1, and LNCaP cells were cultured in RPMI medium supplemented with 10% FBS, 50 U/mL penicillin, and 50 μg/mL streptomycin. Long term androgen deprivation (LTAD) cells were cultured in phenol-red free RPMI medium supplemented with 10% charcoal-dextran stripped FBS, 50 U/mL penicillin, and 50 μg/mL streptomycin. DU145-CR and PC3-CR, aggressive chemotherapy resistant PC ells, were generated by our group^50^. Briefly, cells were cultured with cabazitaxel in a dose escalation manner (up to 3 nM). After several passages, cabazitaxel resistant phenotype was confirmed by cell proliferation assay and xenograft model. For androgen treatment (10 nM DHT), cells were incubated in phenol-red-free RPMI/DMEM medium supplemented with 2.5% charcoal-dextran stripped FBS for three days before androgen stimulation. Two CRPC patient-derived cells (PDCs) were generated by culturing tumor cells collected from pleural effusion (Patient #1) and circulating tumor cells collected from blood (Patient #2). The cells were cultured on ultra-low attachment culture dishes (Corning, Corning, NY) and grown in StemPro hESC SFM-Human Embryonic Stem Cell Culture Medium (ThermoFisher, Carlsbad, CA) supplemented with 8.8 ng/mL basic FGF (Invitrogen, Carlsbad, CA), 20 μM Y-27632 (Wako, Osaka, Japan), 50 ng/ml EGF (Sigma, St. Louis, MI), 1x B27 additive (Invitrogen), 0.1 nM DHT (Sigma), 0.5 μM A83-01 (Tocris, Bristol,UK), 10 μM SB202190 (Sigma), 1:100 v/v Primocin (Invitrogen), 50 U/mL penicillin, and 50 μg/mL streptomycin. Spheroids were passaged by dissociating with Accumax (Stem Cell, Vancouver, Canada) once a month. Cells were stably maintained for >6 months.

### Reagents and antibodies

Rabbit polyclonal anti-HA (Y-11; 1:200 dilution), rabbit polyclonal anti-AR (H-280; 1:2000 dilution), mouse monoclonal anti-AR (sc-7305; 1:200 dilution), and goat polyclonal anti-GAPDH (V-18; 1:1000 dilution) were purchased from Santa Cruz Biotechnology (Dallas, TX). Rabbit polyclonal anti-FOXA1 (ab23738; 1:2000 dilution), rabbit polyclonal anti-AR-V7 (ab198394; 1:200 dilution), rabbit polyclonal anti-OCT4 (ab181557/ab19857; 1:200 dilution), rabbit polyclonal anti-AcH3K27 (ab177178), rabbit polyclonal anti-K4me1 (ab8895), and mouse monoclonal anti-NRF1 (ab55744; 1:500 dilution) were purchased from Abcam (Cambridge, UK). Mouse monoclonal anti-β-actin (A5441; 1:1000 dilution) was purchased from Sigma. Mouse monoclonal anti-Flag (012-22384; 1:2000 dilution) was purchased from Wako (Tokyo, Japan). Rabbit polyclonal anti-FOXM1(C15410232; 1:500 dilution) was purchased from diagenode (Liège, Belgium). Rabbit polyclonal anti-K4me3 (07-473) and rabbit polyclonal anti-RNA polII (05-623) were purchased from Millipore (Burlington, MA). Rabbit polyclonal anti-NRF2 (61600; 1:500 dilution) was purchased from Active Motif (Carlsbad, CA). Mouse monoclonal anti-TRIM25 (610570; 1:500 dilution) was purchased from BD Biosciences (San. Diego, CA). Secondary antibodies were purchased from Amersham (Little Chalfont, UK, NA931, and NA934: Western blot analysis; 1:5000 dilution), BIO-RAD (Hercules, CA, STAR209P: Western blot analysis following immunoprecipitation; 1:300 dilution) and Jackson ImmunoResearch (West Grove, PA, 115-035-174: Western blot analysis following immunoprecipitation; 1:10000 dilution). Cabazitaxel and Dihydrotestosterone (DHT) was purchased from Wako. Docetaxel (01885), 1,6-hexandiol (HD) (240117), 2,5-hexandiol (HD) (H11904), biotinylated isoxazole (900572), and ribavirin (R9644) were purchased from Sigma. Phospho-ribavirin (NU-1105) was purchased from Jenabioscience (Jena, Germany).

### Human PC tissues

Human formalin-fixed paraffin-embedded primary (*N* = 159) and CRPC/NEPC (*N* = 16) tissues were collected from Keio University Hospital. All patients provided written informed consent to obtained tumor samples. All tissue sections were reviewed by pathologists to confirm PC origin. In addition, NEPC loci were determined by specialized pathologists. The study was approved by the Human Genome, Gene Analysis Research Ethics Committee of the Tokyo Metropolitan Institute of Gerontology (#28-5961), and Keio University (#2016-0084).

### PDC spheroid viability assay

Three thousand Accumax-dispersed single cells per well were seeded in ultra-low attachment plates (Corning). To examine the effect of ribavirin and chemotherapy on spheroid formation, 100 μM ribavirin, 10 nM docetaxel, 10 nM cabazitaxel or vehicle was added to the medium. Spheroid cell viability was evaluated using Cell titer-Glo 3D Cell Viability Assay kit (Promega, Madison, WI) using an automatic luminometer (*N* = 4). Chemiluminescence values were normalized with the vehicle-treated samples.

### Microarray

To characterize the transcriptional program regulated by OCT4 and NRF1, we performed microarray analysis of PC cells (LNCaP, 22Rv1, DU145-CR, parental DU145, and PC3-CR after 72 h of being transfected with siControl, and two siRNAs targeting OCT4 or NRF1. RNA samples were validated to be high quality (RNA integrity score >9.0) by using RNA bioanalyzer (Agilent, Waldbronn, Germany). For gene expression microarrays, the GeneChip Human Clariom S Array (Affymetrix, Santa Clara, CA) was used in accordance with the manufacturer’s protocol. Data analysis was performed using the Affymetrix Microarray Suite software. To compare arrays, normalization was performed on data from all the probe sets. Kyoto Encyclpedia of Genes and Genomes (KEGG) pathway enrichment analysis was performed using the Database for Annotation, Visualization and Integrated Discovery (DAVID 6.7, http://david.ncifcrf.gov/).

### RNA-seq

To characterize the expression profile regulated by ribavirin treatment, we performed RNA sequencing of 22Rv1 cells after 72 h of being treated with ribavirin (10 μM) or vehicle. High quality total RNA samples (RNA integrity scroe >9.0) were subjected to poly-A-selected sequencing library preparation using TruSeq RNA Library Preparation Kit v2 (Illumina, San Diego, CA). The libraries were sequenced by Hiseq 2500 (Illumina) to generate 50 bp single-end reads. To remove rRNA sequences, we used Bowtie 2v. 2.2.6. Mapping to human genome (hg19) was performed using TopHat 2.1.0. Alignments were generated in the SAM format from given single-end reads. For expression analysis, the reads were mapped to the human RefSeq mRNA database. The expression levels of the mapped transcripts were normalized to reads per kilobase of exon per million mapped reads (RPKM) to facilitate comparison among different samples.

### ChIP and ChIP-seq

ChIP and quantitative PCR (qPCR) were performed as previously described^59^. In brief, chromatin from crosslinked PC cells was sonicated, pre-cleared and incubated with corresponded antibody overnight and precipitated with protein G-sepharose (GE healthcare, Chicago, IL). The DNA–protein antibody complexes were washed with Radioimmunoprecipitation assay (RIPA) buffer, Litium buffer, and Tris-EDTA (TE) buffer. Cross-linkage of the co-precipitated DNA-protein complexes was reversed and immunoprecipitated DNA was ethanol precipitated. The fold enrichment relative to input was measured by performing qPCR, using KAPA SYBR Green PCR master mix (Sigma genosys, Tokyo, Japan) and the ABI StepOne system (ThermoFisher). We performed AR, OCT4, K4me1, K4me3, FOXA1, FOXM1, and AcH3K27 ChIP-seq in 22Rv1cells and OCT4, NRF1, NRF2, AcH3K27 in DU145 cells using an Illumina HiSeq 2500 (Illumina, San Diego, CA) as performed in previous studies^5,60^. All sequence libraries were made according to Illumina’s instructions. Unfiltered single-end sequencing of 50 bp was conducted for all samples. The sequenced reads obtained were aligned to the human reference genome (hg19) using Bowtie. Signal scores of TF bindings were calculated using model-based analysis of ChIP-seq (MACS)^61^ v1.4.2 and the threshold for the binding sites was set as *P* < 1.0 E-4. An integrative genomic viewer was used for visualization as previously described^5,60^. Motif discovery (±50 bp around peaks obtained by ChIP-seq) was conducted with the HOMER^31^. To identify superenhancers, we used ranking of superenhancers (ROSE)^33,57^ downloaded from Young Lab (http://younglab.wi.mit.edu/super_enhancer_code.html). NGSPLOT (v.2.47.1) was used to analyze ChIP-seq tag distributions around the peaks. Heat signatures of ChIP-seq signaling were obtained to the left and right of each peak center of the AR bindings using HOMER and NGSPLOT.

### Immunohistochemistry

Immunohistochemistry analyses were performed on PC formalin-fixed paraffin-embedded tissue sections from human samples. Tissues sections were deparaffinized and submitted to immunohistochemistry procedures using the Histofine kit (Nichirei, Tokyo, Japan), which employs the streptavidin-biotin amplification method. Antigen retrieval was performed by heating the slides in an autoclave at 120 °C for 5 min in citric acid buffer (2 mM citric acid and 9 mM trisodium citrate dehydrate, pH 6.0). The antigen-antibody complex was visualized with a 3,3′-diaminobenzidine solution (1 mM 3,3′-diaminobenzidine, 50 mM Tris-HCl buffer, pH 7.6, and 0.006% H_2_O_2_). In immunohistochemical analysis, immunoreactivity (modified H-score) was determined by multiplying intensity score (Negative: 0, Weakly positive: 1, Strongly positive 2) by quantified rate of positive cells per total counted cells (0–100 %) in each case of localized PC (*N* = 143), locally invasive PC (*N* = 16), and CRPC samples (*N* = 16). H-score was determined by specialized pathologists. We determined that AR expression level is low when H-score is less than the median value.

### Immunofluorescence microscopy

Cells cultured on 12-mm circular coverslips (Matsunami Glass, Osaka, Japan) in 24-well plates were fixed with 4% paraformaldehyde for 10 min at room temperature. Cells were then permeabilized with 0.5% Triton X-100/PBS for 2 min. After washing cells with PBS, we blocked cells in Blocking One (Nacalai, Tokyo, Japan) for 30 min. Cells were incubated with anti-OCT4, AR, and NRF1 antibody in PBS overnight at 4 °C. Then, cells were washed three times with PBS and incubated with anti-mouse IgG conjugated to Alexa Fluor 546 (Life Technologies) in PBS for 1 h. Nuclei were counterstained with 4′, 6-diamidino-2-phenylindole (DAPI). Cells were washed 3 times with PBS, coverslips were mounted in glycerol, and cells were visualized using a confocal laser scanning microscope (FV10i; Olympus, Tokyo, Japan).

### Cell proliferation assay

Cells (3 × 103) were plated and cultured in 96-well dishes. CellTiter 96 Aqueous Kit (Promega) an [3-(4,5-dimethylthiazol-2-yl)-5-(3-carboxymethoxyphenyl)-2-(4-sulfophenyl)-2H-tetrazolium, inner salt] (MTS)-based assay was used to measure cell vitality and cell growth rate. The MTS assay was performed in four wells for each group. For PC cell growth assay to evaluate the effect of ribavirin, cells were cultured in 24-well plates at 5 × 10^3^ cells per well. Cells were trypsinized and counted using the trypan blue exclusion method.

### Colony formation assay

Clonogenic assays in response to cabazitaxel treatment were performed by plating 10^4^ cells in 60 mm culture dishes. After 24 h incubation, cells were treated with vehicle controls or with drugs for 72 h before medium was changes. Culture medium was changed freshly every 2–3 days. After 14 days incubation, cell culture dishes were washed with PBS, stained with a 2% crystal violet 10% formalin solution and formed colonies counted by using microscopy.

### Transfection

Cells were transfected with control siRNA or siRNAs (10 nM) targeting AR (s1538), FOXM1 (s5248, s5250), FOXA1 (HSS104878NRF1), OCT4 (s10872, s10871) (Thermo Fisher, Waltham, MA), and NRF1 (RNAi. co., Tokyo, Japan). Designed siRNA sequences were as followed: siRNA *NRF1* #1 S:GGCAAAUGUCCGGAGUGAUGU AS:AUCACUCCGGACAUUUGCCCA, siRNA *NRF1* #2 S:CCACAGCCACACAUAGUAUAG AS:AUACUAUGUGUGGCUGUGGCC. The transfection reagent Lipofectamine RNAiMAX (Thermo Fisher) was used according to the manufacturer’s instructions and cells were incubated for 48–72 h post-transfection. To establish stable cells overexpressing OCT4, we transfected pcDNA3.0-OCT4 or empty vector into DU145 cells using X-tremeGene HP DNA Transfection Reagent (Roche Applied Science, Penzberg, Germany). Clones expressing OCT4 were selected by incubating cells in the presence of G418 (Sigma, St. Louis, MO) at the concentration of 500 μg/mL.

### Xenograft model

Prostate cancer 22Rv1 and DU145-CR cells, suspended in 100 μL medium were mixed with 100 µL of Matrigel (BD Biosciences, San Jose, CA) and subcutaneously implanted into one side of twenty 5-week-old male BALB/c nude mice (CLEA Japan, Tokyo, Japan) using a syringe fitted with a 26G needle. After 7–10 days post-injection, the primary tumor measured ~100 mm^3^. At this point, we performed castration to deprive androgen in the mice bearing 22Rv1 xenografts. These castrated mice were randomly divided into two groups (*N* = 6). Then we started intrapleural injection of 50 mg/kg ribavirin or vehicle (sterile water) daily. Mice bearing DU145-CR xenografts were randomly assigned to four groups (*N* = 5). Then mice were treated with (1) ribavirin (50 mg/kg; daily) + cabazitaxel (5 mg/kg; once a week), (2) vehicle (sterile water; daily) +  cabazitaxel (5 mg/kg; once a week), (3) ribavirin (50 mg/kg; daily) + vehicle (saline solution; once a week), or (4) vehicle (sterile water; daily) + vehicle (saline solution; once a week). Tumor dimensions were monitored using a caliper. Tumor volume was determined according to the formula 1/2 × a × b^2^ (a and b represent the minimal and maximal diameter of tumor, respectively). After monitoring the tumor size for two weeks, the mice were sacrificed and the tumor samples were analyzed. Animal care was in accordance with the Tokyo Metropolitan Institute of Gerontology animal experiment guidelines. The ethics committee of animal experiments at the Tokyo Metropolitan Institute of Gerontology approved our study protocol.

When mice were sacrificed, blood samples for serum biochemical analysis (Oriental Kobo, Shiga, Japan) were collected from hearts. Tumors were homogenized in and RIPA buffer with protease inhibitor cocktail (Nacalai) for western blot analyses. For immunohistochemistry, tumors were formalin fixed and embedded in paraffin. To evaluate the toxicity, body weights for every mouse were recorded every week.

### Immunoblots and immunoprecipitation

Whole cell extracts were prepared in lysis buffer (50 mM Tris-HCl [pH 8.0], 150 mM NaCl, 1% NP-40, peotease inhibitor cocktail (Nacalai)). Protein concentration was determined by performing the bicinchoninic acid (BCA) assay (Pierce, Tokyo, Japan). Obtained lysates were loaded on SDS-polyacrylamide gels, separated using electrophoresis, and subsequently electrotransferred onto Immobilon-P Membranes (Millipore, Billerica, MA, USA). Membranes were incubated with the specific primary antibodies at 4 °C overnight, and then incubated with secondary antibodies. Antibody-antigen complexes were detected using Western Blotting Detection Reagents (Pierce, Tokyo, Japan). For immunoprecipitation, extracts were incubated with the indicated antibodies overnight at 4 °C. Following 2 h of incubation with protein G agarose (GE healthcare), beads were washed three times with lysis buffer, resuspended in 1x Sample buffer (Nacalai) and boiled for 5 minutes.

### Biotynylated isoxazole (b-isox)-mediated precipitation

Cell pellets were resuspended in 1 mL lysis buffer (20 mM Tris-HCl (PH 7.4), 300 mM NaCl, 5 mM MgCl_2_, 1% NP40, 10% glycerol, 20 mM β-mercaptoethanol, 1x phosphatase inhibitor (Nacalai), RNase inhibitor, and Protease inhibitor (Nacalai)). Sonicated briefly (30 s on, 30 s off, five cycle) and incubated with rotation for 30 min at 4 °C. Protein supernatant was collected by centrifugation at 4 °C, 16,500 × g for 15 min. Then, 5% lysates were saved for input cell extract and remaining were aliquoted equally and incubated with various concentrations (30 and 100 μM) biotinylated isoxazole (Sigma, no. 900572) at 4 °C for 1 h with rotation. Precipitates were isolated by centrifugation at 4 °C, 16,500 × *g* for 15 min. Supernatant was saved and pellets were washed with lysis buffer with protease and RNase inhibitor. Protein was denatured by heating at 98 °C for 5 min with 1x sample buffer for western blot analysis.

### Luciferase reporter assay

LNCaP and 293T cells were plated in 24-well dishes at a density of 3 × 10^4^ cells/well in phenol-red-free medium with 5% charcoal-stripped FBS. The cells were transfected with the vectors. Transfection mix was prepared by combining luciferase reporter plasmids, Renilla luciferase control, and Tk-pRL vector (Promega, Madison, WI) as a reference. Transfection was performed using X-tremeGene HP DNA Transfection Reagent (Sigma). After 24 h of incubation, the cells were treated with 10 nM DHT or vehicle (0.1% ethanol) for further 24 h. Luciferase activity was measured using the Dual Luciferase Assay Kit (Promega) in an automatic luminometer. The firefly luciferase activity was normalized to the Renilla luciferase activity.

### Gel shift assay

The gel shift assay was performed by using the DIG Gel shift kit (Roche) with DIG-labeled oligonucleotides (Supplementary Table 1) containing OCT4-binding peak positions identified by ChIP-sequence. Nuclear lysates were separated using hypotonic buffer (20 mM HEPES [pH 7.9], 10 mM KCl, 1 mM EDTA, 1 mM EGTA, 0.65% NP-40, 1 mM DTT) and RIPA buffer. After mixing probes with nuclear extracts at room temperature, samples were analyzed on a 6% polyacrylamide gel electrophoresis (PAGE) gel. Following transfer to a positively charged nylon membrane (Roche), signals were detected by using anti-DIG antibody. For the supershift experiments, 1 μg specific antibody was added before electrophoresis and incubated for 15 min.

### RNA extraction and qRT-PCR

RNA was isolated using the ISOGEN II (Nippon Gene, Tokyo Japan) in accordance with manufacturer’s instructions. Complementary DNA was synthesized from equivalent concentrations of total RNA using Prime Script (TAKARA bio, Kyoto Japan) in accordance with manufacture’s protocols. Amplification was performed in a StepOne PCR System (Thermo Fisher, Waltham, MA) using KAPA SYBR Green (Sigma). Fold changes for experimental groups relative to the loading control were calculated by ΔΔCt method. Primer sequences were provided in the Supplementary Table 2.

### Fluorescence recovery after photobleaching

Cells were plated in glass-bottom microwell dishes (Matsunami Glass). 293T cells transfected with Venus-FOXA1, Venus-AR, Venus-NRF1, with or without mCherry-OCT4 were cultured in hormone-deprived media for 48 h. LNCaP cells transfected with Venus-AR or mCherry-OCT4 were cultured in hormone-deprived media for 3 days. 22Rv1 cells were treated with siControl or siOCT4 #1. After 48 h incubation, cells were transfected with Venus-AR. The cells were treated with 10 nM DHT for 2 h. Dishes were placed in humidity-controlled chamber set at 37 °C with 5% CO_2_. The FRAP experiments were conducted using the FRAP wizard on a TCS SP8 confocal microscope (Leica, Wetzlar, Germany). Images were collected using a ×100 Oil immersion before and after bleaching using 488 nm line from a white laser with a power intensity at set at 5–10% in the software. Photobleaching was performed in small region within the nucleus (2 μm × 2 μm) using the 488 nm laser line at 100% intensity for 5 cycles at 1 s intervals. Fluorescence recovery was monitored in images taken during 20 s at 0.8 s intervals immediately after photobleaching followed by images taken for 150 s at 10 s intervals. Data were analyzed in Prism 6 (Graphpad Software, La Jolla, CA) for 5–10 cells per condition.

### Construction

Coding sequences of *OCT4*, deleted mutants of *OCT4*, *NRF1*, *FOXA1*, and *AR* were amplified and cloned into expression vector pcDNA3.0 (Flag tagged or HA-tagged). Venus-Flag-tagged pcDNA3 was synthesized by amplifying Venus sequences from USE10C/pCS2 (RIKEN BRC DNA BANK) and subcloning them into pcDNA3.0 with Flag tag at C-terminal. Flag-mCherry-pcDNA3 was synthesized by amplifying mCherry sequences from pET-mCherry (RIKEN BRC DNA BANK) and subcloning into pcDNA3.0 with Flag tag at N-terminal. Then Venus-Flag-tagged *AR*, *FOXA1*, and *NRF1* were synthesized by cloning coding sequences from cDNA of PC cells to EcoRV/XhoI restriction site. Flag- mCherry-tagged OCT4 was synthesized by subcloning OCT4 coding sequence to EcoRI/EcoRV restriction site. To construct luciferase vectors of OCT4BSs, each enhancer region was amplified by PCR and cloned into pGL3-SV40-promoter vector (Promega) using MluI/XhoI site. Plasmid DNA was transformed into competent cells for amplification and purified using Maxiprep Plasmid extraction kit (Qiagen).

### In vitro binding assay

Full-length *OCT4* and *OCT4* truncation mutants were cloned into HA-tagged pcDNA3.0 vectors. Full-length *AR*, and *FOXA1* were cloned into Flag tagged pcDNA3.0 vectors. After cloning, the fusion proteins were overexpressed in 293T cells by transfection. 293T ells were treated with 10 nM DHT or vehicle for 24 h. Proteins were purified by using Flag (M2) agarose beads (Sigma) and HA agarose beads (Wako). The slurry was centrifuged at 1500×*g* for 1 min. The resin pellets were washed three times with 1 mL lysis buffer, followed by centrifugation. Protein was eluted twice with 100 μL of elution buffer (0.1 mol/L Glycine-HCl (pH2.4)) followed by addition of 10 μL 1 M Tris-HCl buffer (pH10.4). To perform in vitro binding experiments^62^ with some modifications, purified proteins were incubated in binding buffer (20 mM Tris-HCl (pH 7.5), 150 mM NaCl, 0.1% Triton X-100, 1 mM dithiothreitol, and 1x protease Inhibitor Cocktail (Nacalai) and incubated at 4 °C for 5 h. Ribavirin-phosphate, an activated metabolite of ribavirin in cells, was used to analyze the effect of ribavirin in vitro. FOXA1 was then immunoprecipitated and bound proteins were analyzed by western blotting as described above.

### Droplet assay

For protein expression, plasmids were transfected to 1.0 × 10^7^ 293T cells. After 24 h incubation, cells were treated with 10 nM DHT or vehicle. Followed by additional 24 h incubation, cells were collected and resuspended and sonicated (three cycles of 30 sec) in NP-40 buffer (50 mM Tris-HCl [pH 8.0], 150 mM NaCl, 1% NP-40, peotease inhibitor cocktail (Nacalai)). The lysates were cleared by centrifugation at 12,000 × *g* for 30 min and added to 100 μL of Flag (M2) agarose beads (Sigma) that had been pre-equilibrated with 10 volumes of the same buffer, and rotate at 4 °C overnight. The slurry was centrifuged at 1500 × *g* for 1 min. The resin pellets were washed three times with 1 mL NP40 buffer, followed by centrifugation. Protein was eluted twice with 100 μL of elution buffer (0.1 mol/L Glycine-HCl (pH2.4)) followed by addition of 10 μL 1 M Tris-HCl buffer C (pH10.4). Purified proteins were dialyzed against two changes of buffer containing 50 mM Tris 7.5, 125 mM NaCl, 10% glycerol, and 1 mM DTT at 4 °C. Proteins were further concentrated using Amicon Ultra centrifugal filters (30 K MWCO or 50 K MWCO, Millipore). Protein concentration was determined by BCA assay. Equal amount of proteins was added to solutions at varying concentrations with 125–500 mM NaCl and 10% PEG8000 as crowding agent in droplet formation buffer (50 mM Tris-HCl pH 7.5, 10% glycerol, 1 mM DTT). The protein solutions were immediately loaded onto glass slides with coverslips. Slides were then imaged with FV10i confocal microscope with a ×60 objective.

### Statistics and reproducibility

Each experiment was performed at least two times from distinct samples. We performed OCT4 ChIP-seq in biological replicates. Results in Figs. 1c, 5i, and 8h, j, and Supplementary Figs. 5b and 11e are representative data of two independent experiments. Results in Figs. 2d, e, 4a, 6d, f, 7a, c, and 8a, b, and Supplementary Figs. 1e, g, 3a, 5e, f, 6d–f, 8c, 9b, and 11a are representative data of three independent experiments. In these repeated experiments, we observed similar results. Data are expressed as the mean ± SD. The unpaired Mann–Whitney *U* test and two-sided Student’s *t* test were used to determine differences between means of groups. Significance was defined as *P* < 0.05. In the experiment of the stable cell lines, we performed two-way analysis of variance (ANOVA) to determine the significance between two groups. To analyze correlations, we used Spearman’s correlation tests. We used Dunnett’s test following one-way ANOVA was used to determine the difference of multiple samples. Survival analyses were performed using the Kaplan–Meir method and curves were compared by the log rank test. Other statistical tests were described in the figure legends. Excel v16.16.27 (Microsoft, Redmond, WA) or GraphPad Prism software ver. 6.0 was used for the statistical analysis.

### Reporting summary

Further information on research design is available in the Nature Research Reporting Summary linked to this article.

## Supplementary information

Supplementary Information

Reporting Summary

## Data Availability

ChIP-seq, microarray and RNA-seq data have been deposited in the Gene Expression Omnibus (GEO) repository (www.ncbi.nlm.nih.gov/geo) with accession numbers: GSE141806, GSE146656, GSE146886, and GSE123565. RNA-seq data have been deposited in the Japanese Genotype-phenotype Archive (JGA) under accession code JGAS00000000198. Publicly available data downloaded from GEO was GSE35988. We used data of RNA-seq data (Beltran et al.^52^ Multi-Institute, Robinson et al.^53^ SU2C/PCF Dream Team, and Abida et al.^54^ SU2C/PCF Dream Team) in cbioportal (http://www.cbioportal.org/). Source data for Figs. 1–8 and Supplementary Figs. 1–11 are provided with this paper. Other relevant data in this study are available from the corresponding author upon reasonable request. Source data are provided with this paper.

## Source data

Source Data
