# Supplementary material for: Subtype-specific collaborative transcription factor networks are promoted by OCT4 in the progression of prostate cancer
Source: Nat Commun. 2021 Jun 18;12:3766. doi: 10.1038/s41467-021-23974-4 (PMC8213733; doi:10.1038/s41467-021-23974-4)
Supplement: Supplementary file 1 — Supplementary Information [file 41467_2021_23974_MOESM1_ESM.pdf]

Supplementary Table 1. Sequences for gel shift assay

OCT4BS (*RHOU*: FOXA1 motif + ARE)

Fw: CTCATGGTTATTTGCATTCCAAAGAACTGTTTGCTTTAGCTTAGC

Rv: CTGTGCTAAGCTAAAGCAAACAGTTCTTTGGAATGCAAATAACCA

OCT4BS mutated

Fw: CTGTGCTAAGCTAAATTGAAAAGTTCTTTGAAATTTAAATAACCA

Rv: CTCATGGTTATTTAAATTTCAAAGAACTTTTCAATTTAGCTTAGC

OCT4/NRF1#1 (*XRCC5*)

AGAATCTGCGCATGCTCGGAGAGAATCTGCGCATGCTCGGCCGG

GATTCCGGCCGAGCATGCGCAGATTCTCTCCGAGCATGCGCAGA

OCT4/NRF1#2 (*RAD21*)

CCCAGAAGGAGCGGCGCAGGCGCAATGACTATTTCCTTTTC

AAAGAAAAGGAAATAGTCATTGCGCCTGCGCCGCTCCTTCT

Supplementary Table 2. Primer sequences for qPCR analysis

<u>Primers for qRT-PCR</u>

OCT4	Fw: ACACTCGGACCACATCCTTC
	Rv: AGTGAGGCAACCTGGAGA
NRF1	Fw: ATCAGCAAACGCAAACACAG
	Rv: CTGAAGCATCAGCCAATGTG
RAD21	Fw: TGACTTTGATCAGCCACTGC
	Rv: TCTCACGATCATCCATTCCA
XRCC5	Fw: TCGTGAGCATGGATGTGATT
	Rv: GTCCCCACTTCCATCTTCCT
NDUFA3	Fw: CCATTGAGCCCCTACTTCAA
	Rv: TTCCCACCTCTGATCACACA
MLH3	Fw: GTAGCCCTGGGAAGAATGGT
	Rv: TCGGAAGGAAAGGAAGAACA
IGF1R	Fw: CGCTTCCCACACGTGCTT
	Rv: CTGTGAGAAGACCACCATCAACA
RHOU	Fw: CCCAGCTCCCTTCATATTCT
	Rv: GTGGACAAATATGGGAATGAGTTG
GAPDH	Fw: GGTGGTCTCCTCTGACTTCAACA
	Rv: GTGGTCGTTGAGGGCAATG
Myoglobin (MB)	Fw: AAGTTTGACAAGTTCAAGCACCTG
	Rv: TGGCACCATGCTTCTTTTAAGTC

Primers ChIP-PCR

GAPDH	Fw: CTAGCCTCCCGGGTTTCTCT
	Rv: TCGACAGTCAGCCGCATCT
RAD21	Fw: AAATCGGCAGGATGTTTACG
	Rv: GAGAATCACCTTTGCGCTTG
XRCC5	Fw: GGCGGAATGGAGAGAATGT
	Rv: GGCAGATAGCGGAAAGAGC
NDUFA3	Fw: GCTTGCTCCTGACACCACTT
	Rv: CCCTGGAGCACAAGTAGAGG
negative control (N.C)	Fw: CCTGGAGGGCTTGGAGATG
	Rv: GATCCTACGGCTGGCTGTGA
OCT4BS #1 (KLF4) /SE #1	Fw: GCCCCTAAATGGCCCTAATA
	Rv: TCCTCTGGTTCCTCCTCAGA
OCT4BS #2 (FZD5) /SE #2	Fw: TTGTTTTCAGAGACGGCAGA
	Rv: CCACCCTTGAATGAAGTGCT
OCT4BS #3 (RHOU) /SE #3	Fw: CTCTAAGGGCTCTCCCTGGT
	Rv: AATGGGGGTATCATCTTCCA
OCT4BS #4 (IGF1R) /SE #4	Fw: ACCAGCCGCTGAAACATAGT
	Rv: CTGCCGCCCAGACTTATCTA
OCT4BS #5 (NDRG1)	Fw: GGTGGTTCCATAGGGATGTG
	Rv: GCAGAACAAATCCTCCCAAA
OCT4BS #6 (*UAP1*)	Fw: AATTCCCCTTGGGTAACAGC
	Rv: CTAAGCAACCGTGCTAGGTG

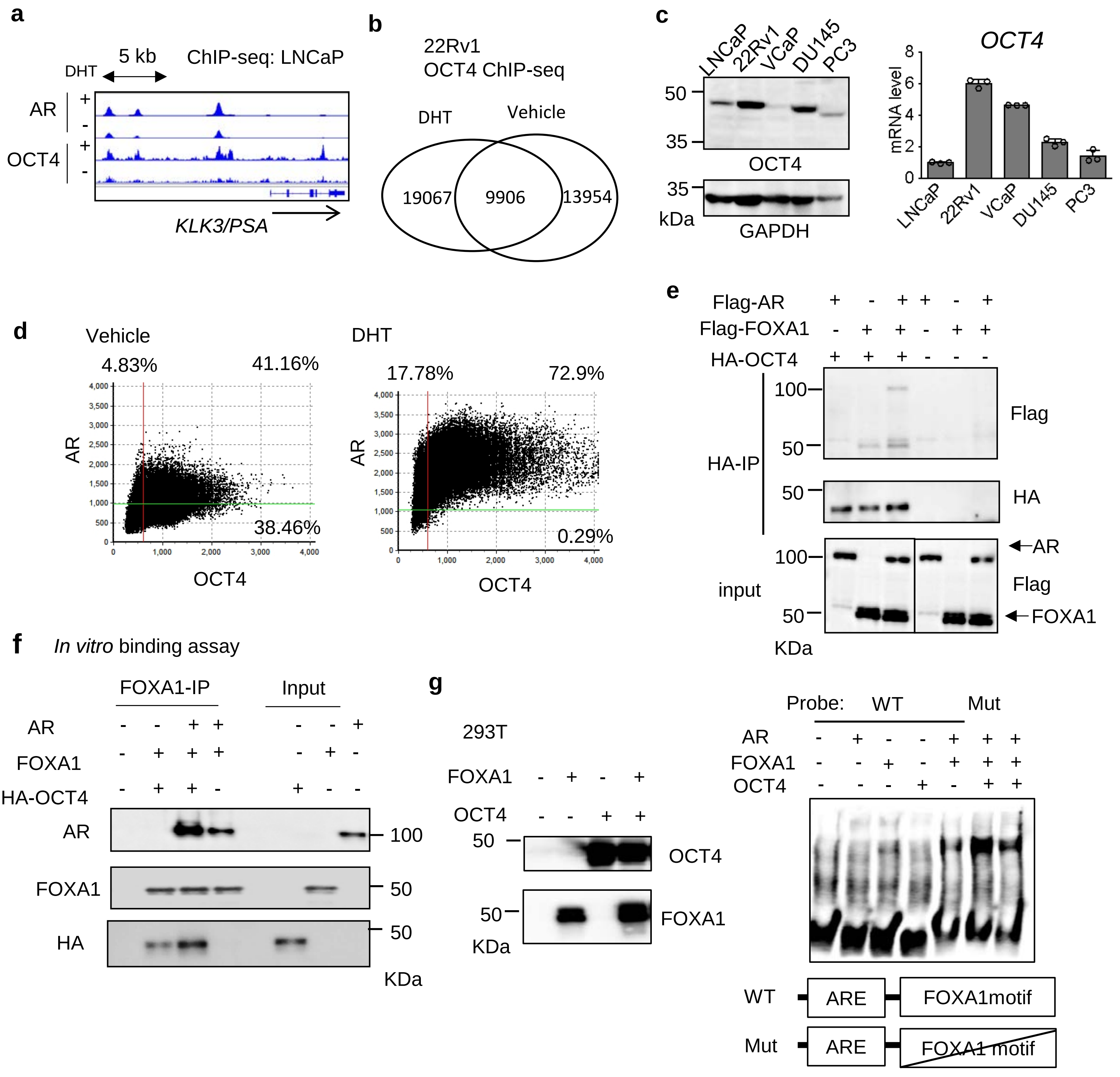

Supplementary Figure 1. Regulation of AR signals and enhancer activity by OCT4-binding in PC ells.
(a) Representative locus of OCT4-binding sites (OCT4BSs) at the enhancer/promoter regions of KLK3/PSA in LNCaP cells. (b) Venn-diagram shows the overlap of OCT4BSs in 22Rv1 cells. Cells were treated with 10 nM DHT or vehicle for 24 h. (c) Expression level of OCT4 in several PC cell lines. Western blotting and qRT-PCR analysis (technical triplicates) were performed. Data are presented as average ± S.D. (d) Quantification of AR and OCT4 immunofluorescence levels (Fig. 2e) in nuclei of 22Rv1 cells. (e) Complex formation of three TFs facilitate the OCT4 interaction with FOXA1 and AR. Immunoblots after immunoprecipitation (IP) with anti-HA antibody in 293T cells transfected with Flag-AR, Flag-FOXA1 or HA-OCT4 as indicated. (f) *In vitro* binding assay using purified proteins (FOXA1, AR, and OCT4) immunoprecipitated by FOXA1-antibody. (g) Increased DNA binding ability of FOXA1/AR complex by OCT4. Nuclear extracts of 293T cells transfected with AR, FOXA1, or OCT4 were used. Two sequences of probes (WT: wild type probe, Mut: FOXA1-motif mutated) were used for EMSA. Source data are provided as a Source Data file.

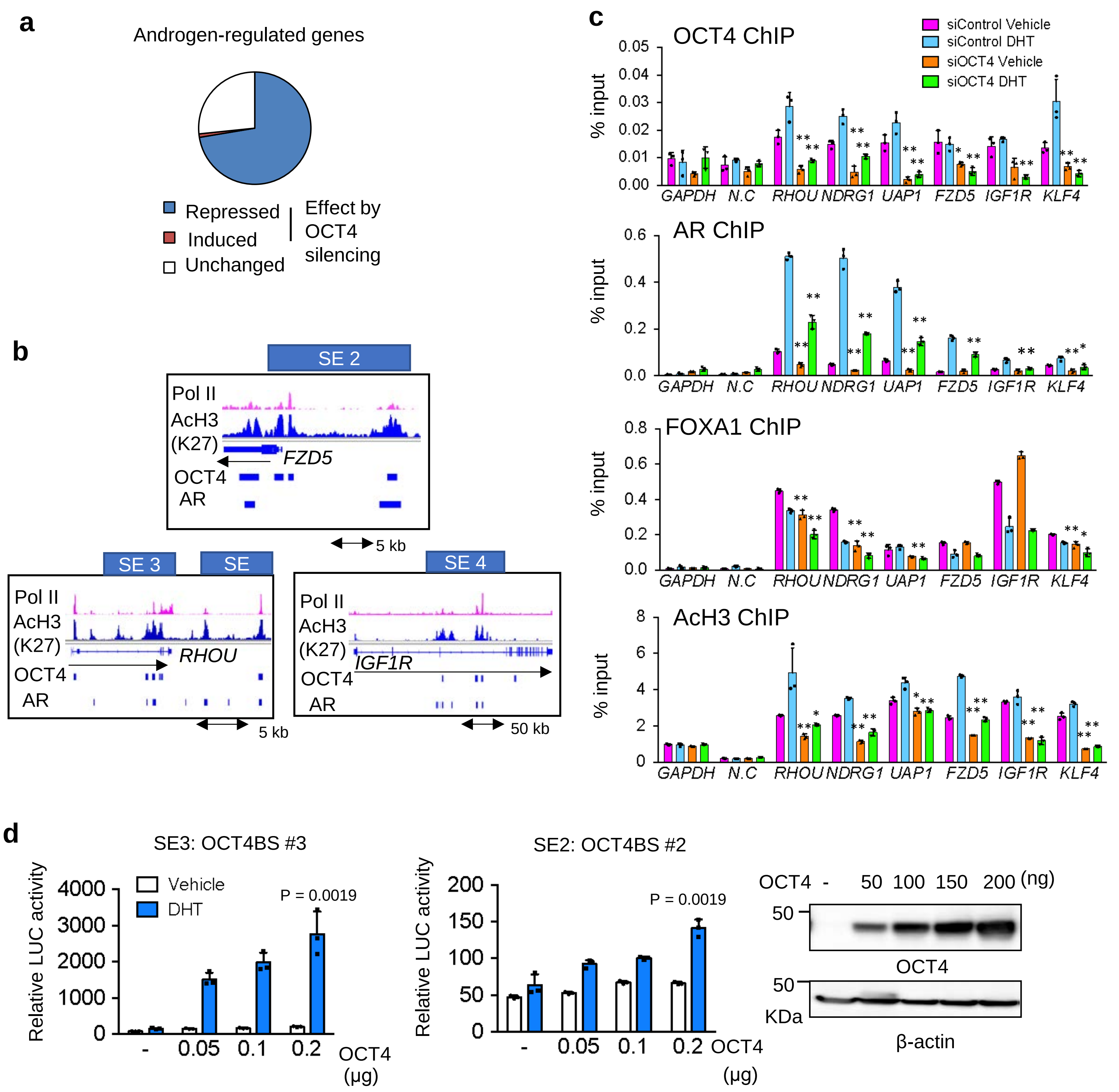

Supplementary Figure 2. The role of OCT4 in superenhancer (SE) establishments to facilitate AR transcription program.
(a) Microarray analysis in 22Rv1 cells transfected with siControl or siOCT4 #1. Cells were treated with DHT or vehicle for 24 h. Androgen-dependent upregulated genes (Fold > 1.5) were identified. Effect of siOCT4 #1 on the responses by DHT. Genes were classified as repressed (Fold < 0.7), induced (Fold > 1.3), or other. (b) Identification of SEs in advanced PC cell model. ROSE analysis of AcH3K27 ChIP-seq signals in 22Rv1 cells. Representative genes in the vicinity of SEs are indicated. Signal tracks of SE signal (AcH3K27), RNA pol II reads on representative SE genes. (c) ChIP assay of OCT4, AR, FOXA1 and AcH3 was performed (N = 3, technical triplicates). 22Rv1 cells were treated with siControl, siOCT4 #1 for 48 h. *P < 0.05, **P < 0.01. Two-sided t-test was performed. Data are shown as average ± S.D. (d) OCT4 addition enhances androgen-mediated enhancer activity of OCT4 binding sequences. LNCaP cells were transfected with luciferase vector including OCT4-binding sequences (#2 and #3 situated in SE #2 and SE #3, respectively). Luciferase assay was performed to analyze the transcriptional activity (N = 3, biological independent samples). Cells were transfected with HA-OCT4 or empty vector as indicated. Immunoblots show overexpression of OCT4 in transfected LNCaP cells. Two-sided t-test was performed. Data are shown as average ± S.D. Source data are provided as a Source Data file.

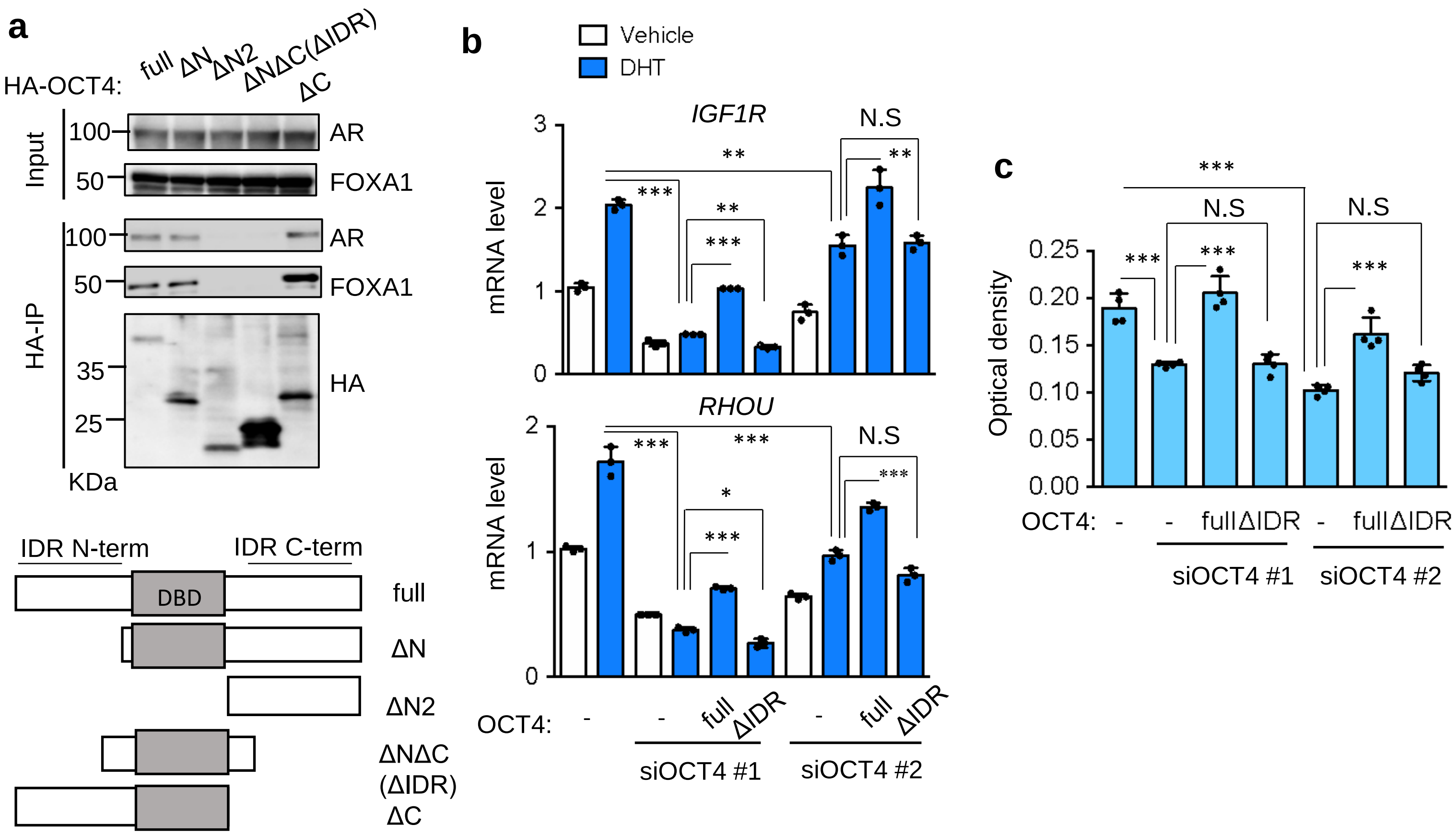

Supplementary Figure 3. OCT4 enhances AR-mediated transcriptional activity through IDR domains.
(a) OCT4 interaction with AR and FOXA1 is dependent on two IDR regions. Four HA tagged deletion mutants of OCT4 were constructed as indicated. 293T cells were transfected with Flag-AR, Flag-FOXA1 and HA-OCT4 mutants. Cells were treated with 10 nM DHT for 24 h before cell harvest. Immunoprecipitation with anti-HA antibody and subsequent immunoblots with indicated antibodies were performed. (b) Downregulation of *IGF1R* and *RHOU* mRNA levels in 22Rv1 cells treated with siOCT4 #1 or #2 compared with siControl were rescued with OCT4-full but not OCT4ΔIDR. qRT-PCR was performed to analyze the androgen-mediated gene inductions (N = 3, technical replicates). N.S: not significant. *P < 0.05, **P < 0.01, **P < 0.001. Two-sided t-test was performed. Data are shown as average ± S.D. (c) OCT4 exhibits activation of AR signals and cell proliferation through IDR domains in OCT4. Growth inhibition in 22Rv1 cells treated with siOCT4 #1 or #2 compared with siControl were rescued with OCT4-full but not OCT4ΔIDR. MTS assay was performed to measure the cell viability (N = 4 , biological independent samples). N.S: not significant. *P < 0.05, **P < 0.01, **P < 0.001. Two-sided t-test was performed. Data are shown as average ± S.D. Source data are provided as a Source Data file.

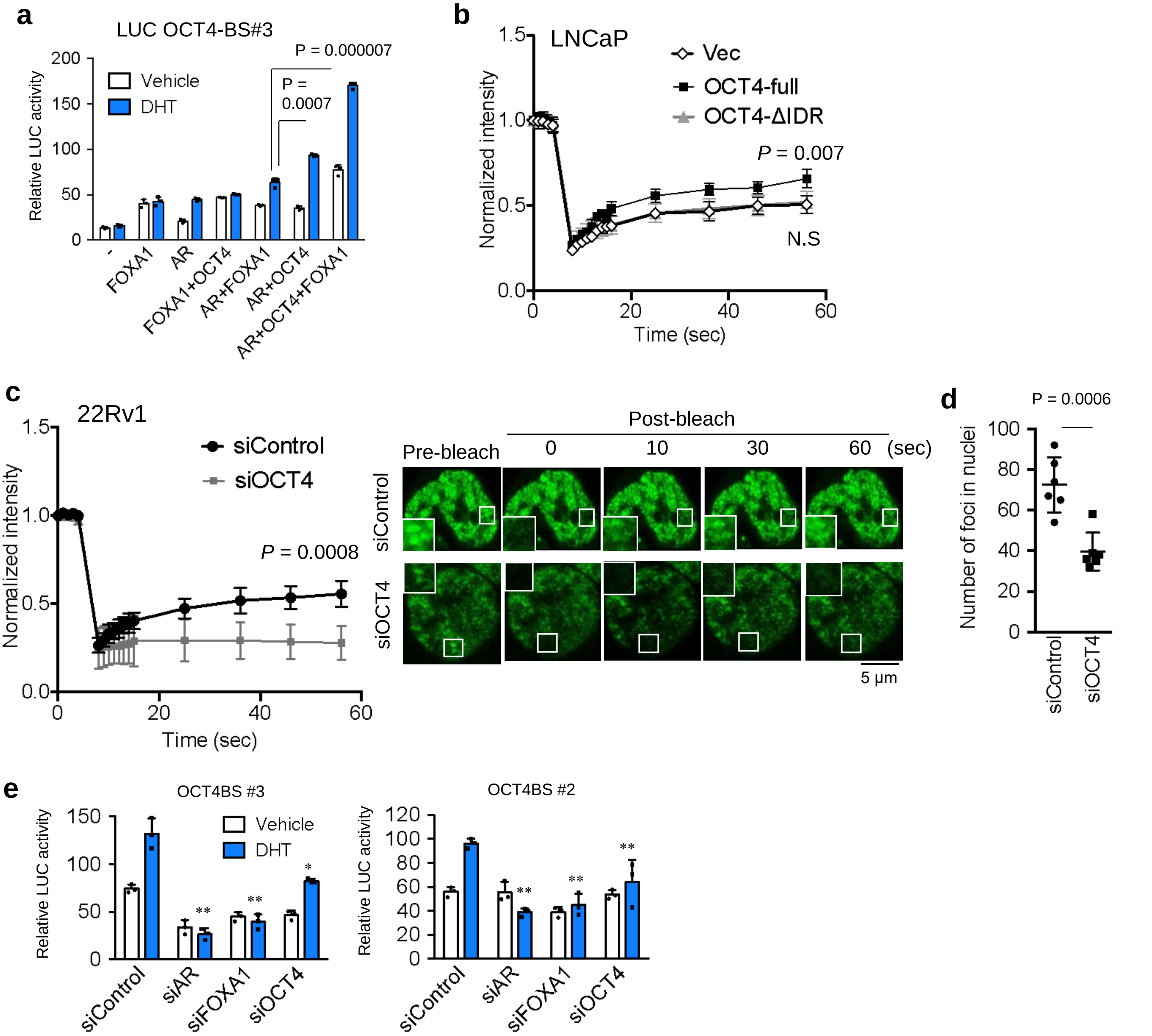

Supplementary Figure 4. OCT4 enhances AR mobility in nuclei to facilitate AR/FOXA1 mediated transcription activity.

(a) OCT4 addition enhances androgen-mediated enhancer activity of OCT4-binding sequences. 293T cells were transfected with luciferase vector including OCT4-binding sequence #3. Luciferase assay was performed to analyze the transcriptional activity (N = 3, biological independent samples). Cells were transfected with AR, FOXA1, OCT4 or empty vector as indicated. Two-sided t-test was performed. Data are shown as average ± S.D. (b) FRAP kinetic plots to analyze the effect of OCT4 in PC cells. Fluorescence recovery was monitors in LNCaP cells transfected with OCT4, OCT4ΔIDR and empty vector (Vec, OCT4:N = 4, OCT4ΔIDR: N = 5, biological independent cells). Cells were treated with DHT for 2 h before assays. Two-sided t-test was performed. Data are shown as average ± S.D. (c) Fluorescence recovery was monitors in 22Rv1 cells transfected with siControl or siOCT4 #1 (N = 5, biological independent cells). Cells were treated with DHT for 2 h before assays. Two-sided t-test was performed. Data are shown as average ± S.D. (d) Quantification of nuclear foci formed in 22Rv1 cells transfected with AR/FOXA1-venus. N=6, biological independent cells. Two-sided t-test was performed. Data are shown as average ± S.D. (e) Loss of AR, FOXA1 and OCT4 repressed androgen-mediated enhancer activity of OCT4 binding sequences. LNCaP cells were transfected with luciferase vector including OCT4 binding sequences (#2 and #3). Luciferase assay was performed to analyze the transcriptional activity (N = 3, biological independent samples). Cells were transfected with siAR, siFOXA1 and siOCT4 #1 as indicated. *P < 0.05, **P < 0.01. Two-sided t-test was performed. Data are shown as average ± S.D. Source data are provided as a Source Data file.

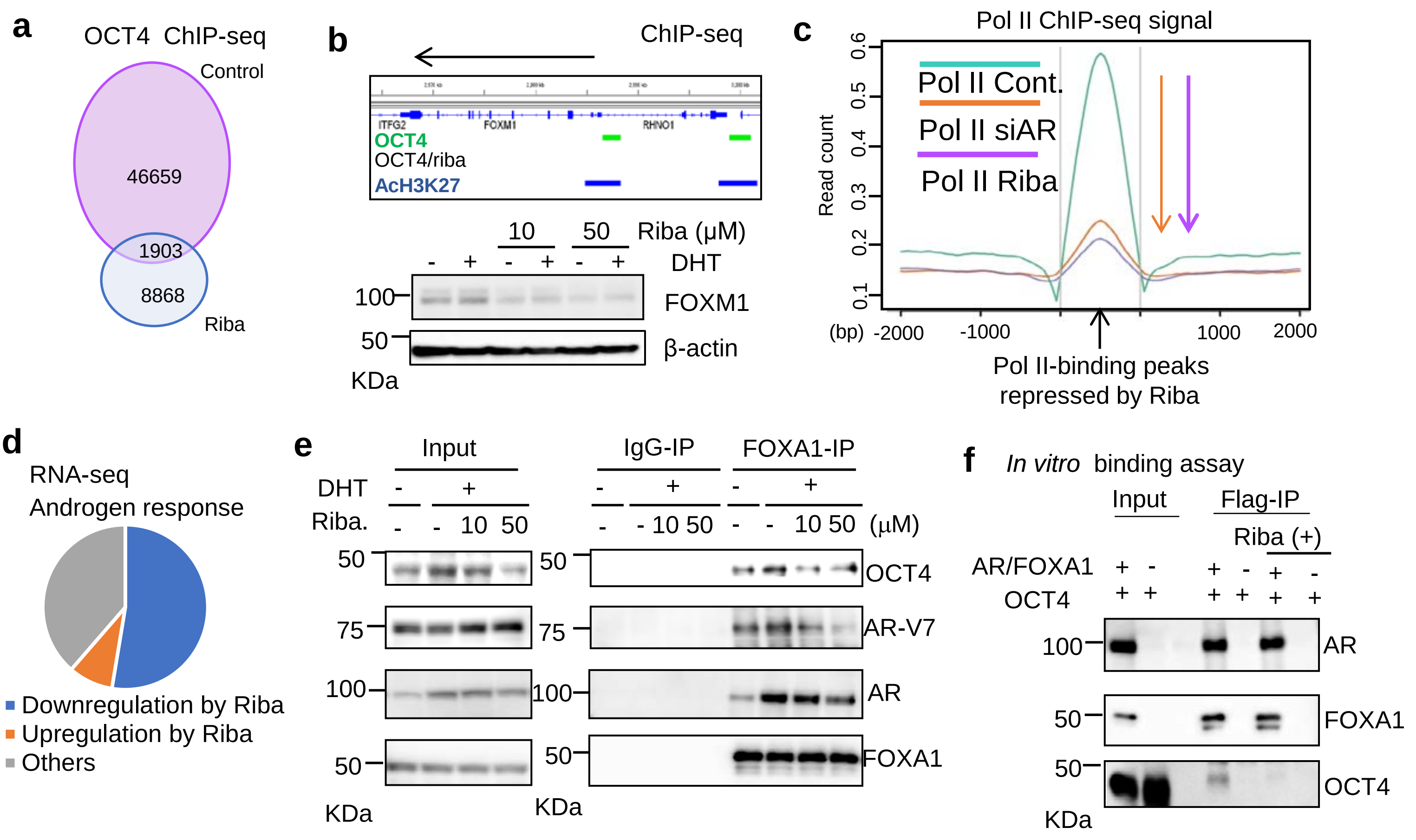

Supplementary Figure 5. Ribavirin treatment repressed AR-occupied enhancer activity.
(a) Venn-diagram showed that OCT4BSs were changed by ribavirin (Riba) treatment. OCT4 ChIP-seq data was used. (b) (Upper) FOXM1 is the representative target gene of OCT4 repressed by ribavirin. ChIP-seq result is shown. (Lower) Immunoblot of FOXM1 in 22Rv1 cells treated with vehicle or ribavirin as indicated. (c) Composite plot shows pol II ChIP-seq signals around pol II-binding sites repressed by ribavirin. (d) Androgen-mediated gene induction was attenuated by ribavirin treatment. Summary of gene regulation by ribavirin treatment. 22Rv1 cells were treated with vehicle or 10 nM DHT for 24 h. Androgen regulated genes (Fold > 1.5) were identified by RNA-seq analysis. We determined androgen response was downregulated (Fold < 0.7) or upregulated (Fold > 1.4). (e) Ribavirin treatment inhibits AR-association with OCT4 and FOXA1. Immunoblots of AR, FOXA1 and OCT4 after immunoprecipitation (IP) by anti-FOXA1 antibody in 22Rv1 cells. Cells were treated with vehicle, DHT or ribavirin as indicated. (f) In vitro binding assay shows the cooperative complex formation of three TFs. Purified proteins (Flag-FOXA1, Flag-AR and HA-OCT4) were mixed with ribavirin-phosphate (10 μM) or vehicle and immunoprecipitated using Flag-antibody. Source data are provided as a Source Data file.

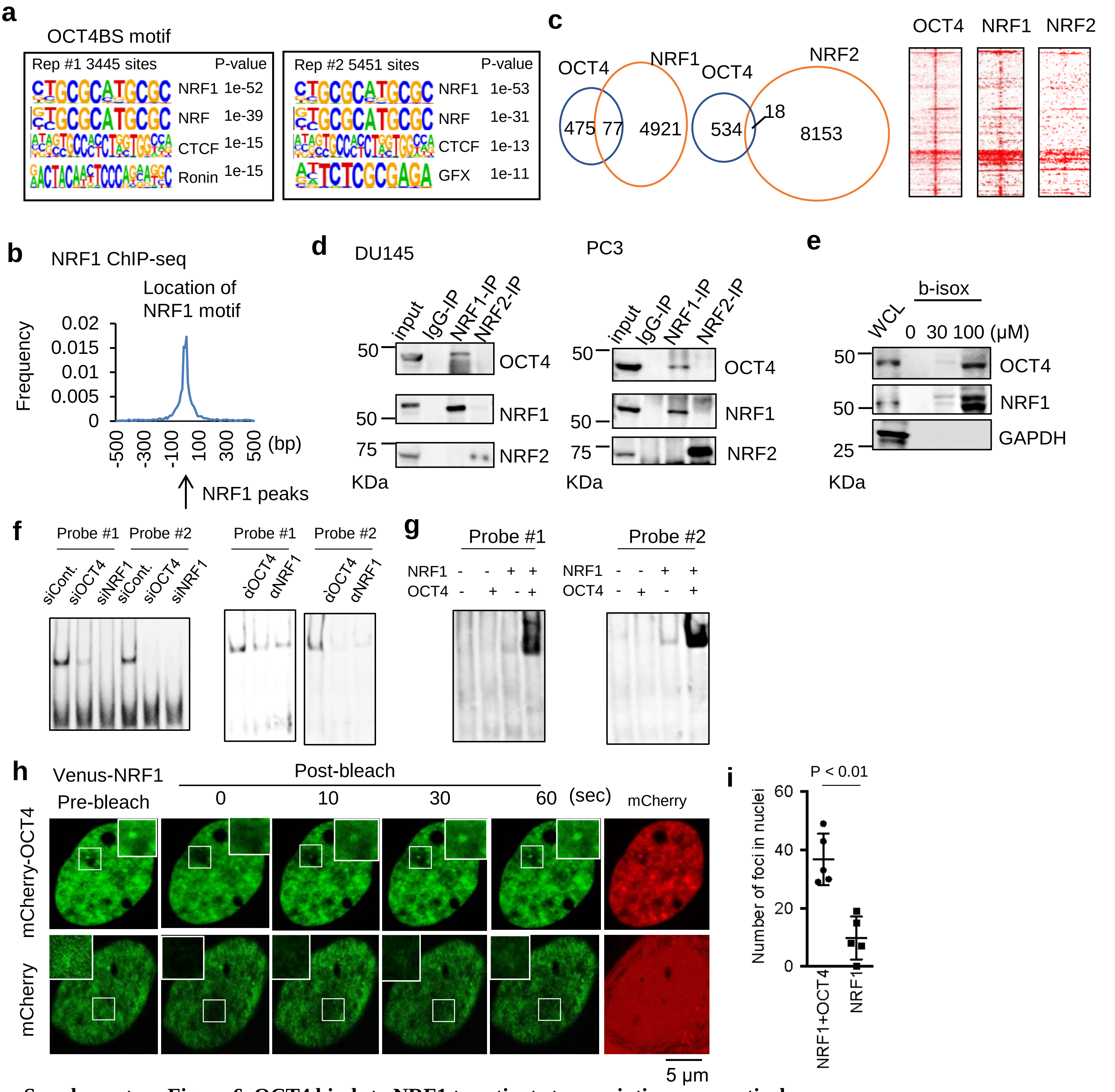

Supplementary Figure 6. OCT4 binds to NRF1 to activate transcription cooperatively.

(a) Global mapping of OCT4BSs in AR-low expressing PC cells. OCT4 ChIP-seq was performed in DU145 cells (N = 2, Rep #1 and Rep #2, biological replidates). Overlapped sites in replicates (573 sites, P < 1E-4) were determined to be OCT4BSs. HOMER motif scan in each ChIP-seq result of significant peak shows that OCT4-binding peaks are enriched in NRF1-binding motif. (b) HOMER analysis of NRF1-binding peaks in DU145 cells. Distribution of NRF1 motif around NRF1 peaks is plotted. (c)Venn-diagram and heatmap show the overlapped regions of OCT4 and NRF1 in DU145 cells. In contrast, low signals and less overlapped regions between NRF2 and OCT4. (d) Interaction of OCT4 with NRF1 in AR-negative PC cells. Immunoblots after immunoprecipitation (IP) by anti-NRF1 and anti-NRF2 antibodies in DU145 and PC3 cells. (e) Immunoblots showing that NRF1 and OCT4 are precipitated by b-isox in DU145 cells. GAPDH was used as a negative control. WCL: whole cell lysate. (f) EMSA to analyze the DNA-binding ability of TF complex including OCT4 and NRF1. Two sequences of OCT4-binding sequences (Probe #1 and Probe #2) were used. Specific antibodies for OCT4 and NRF1 were used to confirm the band include these TFs. (g) DNA-binding ability is regulated by OCT4 and NRF1. Nuclear extracts of 293T cells transfected with OCT4 or NRF1 as indicated were used for EMSA with Probe #1 and #2. (h) FRAP kinetic images from pre- and post-bleaching cells. 293T cells were transfected with Venus-NRF1 or mCherry-OCT4. (i) Quantification of nuclei foci by counting the number of foci (N = 5, biological independent cells). Two-sided t-test was performed. Data are shown as average ± S.D. Source data are provided as a Source Data file.

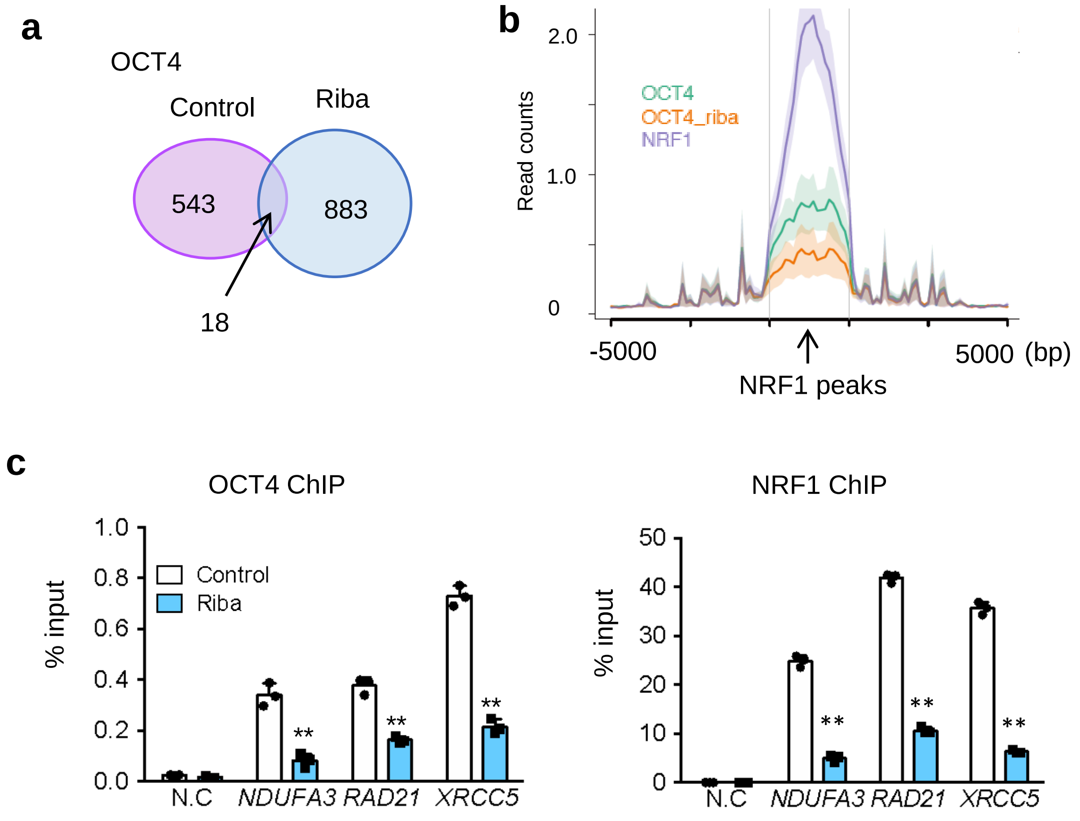

Supplementary Figure 7. Ribavirin treatment repressed OCT4/NRF1 recruitment to the promoter regions.
(a) Venn-diagram shows that OCT4BSs were changed by ribavirin treatment. OCT4 ChIP-seq data in DU145 cells ($P < 1E-5$) was used. (b) Composite plot shows OCT4 and NRF1 ChIP-seq signals around NRF1 binding peaks. Data are shown as mean ± S.E.M. (c) DU145 cells were treated with ribavirin (Riba) (10 μM) or vehicle for 48 h. ChIP analysis was performed using anti-NRF1 antibody (N=3, technical replicates). **$P < 0.01$. Two-sided t-test was performed. Data are shown as average ± S.D. Source data are provided as a Source Data file.

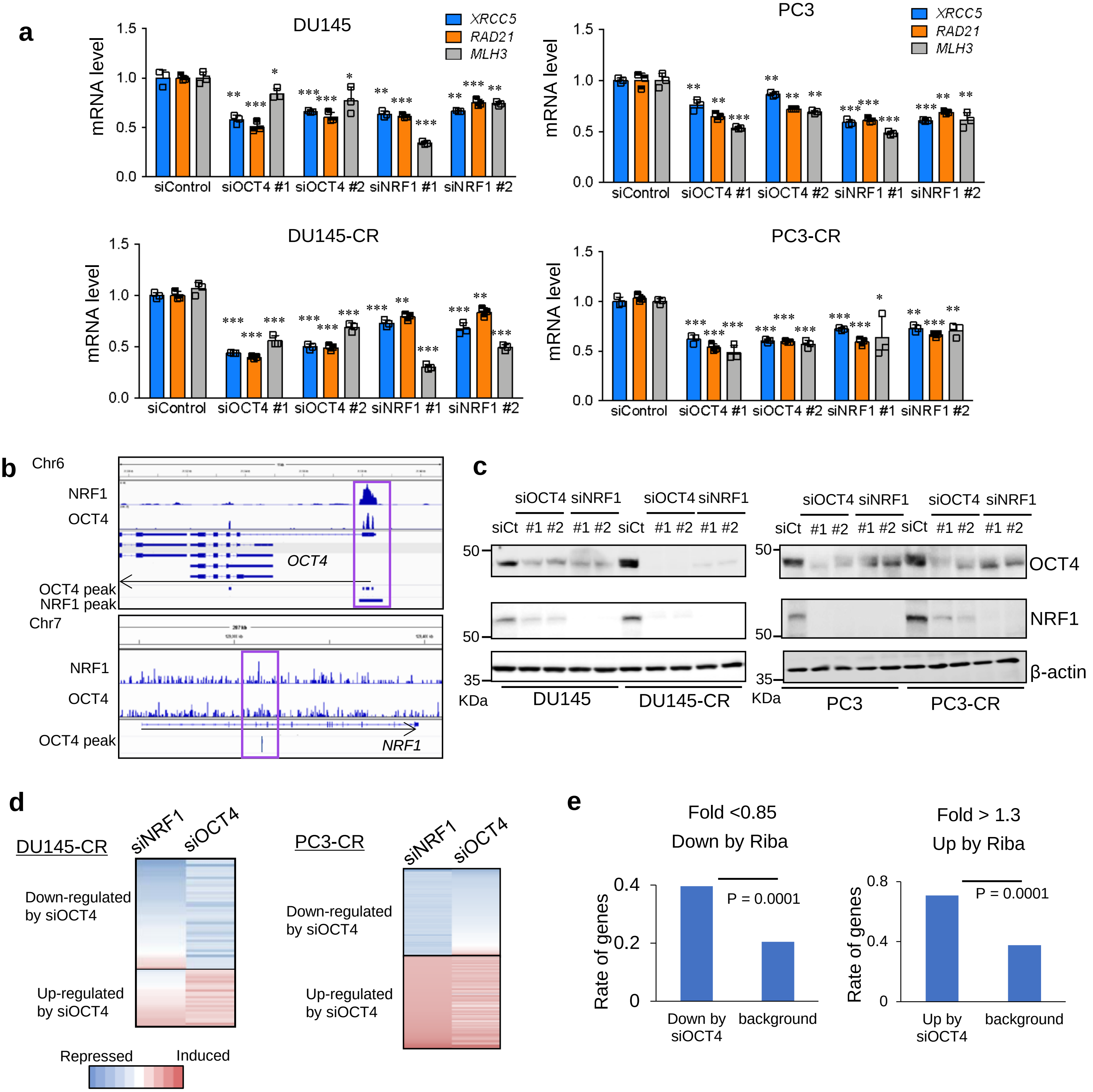

Supplementary Figure 8. The role of OCT4 and NRF1 complex in promoting chemotherapy-resistant properties of PC cells. (a) qRT-PCR analysis to measure mRNA level of OCT4 target genes in DU145-CR, PC3-CR, and their parental cells. More evident repression by siRNA treatment was observed compared with parental cells. *P < 0.05, **P < 0.01, ***P < 0.001. (N=3, technical replicates). Two-sided t-test was performed. Data are shown as average ± S.D. (b) Mutual regulations between OCT4 and NRF1. Signal tracks of NRF1 and OCT4 ChIP-seq reads on OCT4 and NRF1 locus. OCT4- and NRF1-binding regions (P < 1.0E-4 by MACS) were shown by boxes. (c) Immunoblots of OCT4 and NRF1 in DU145-CR, PC3-CR, and their parental cells. Cells were treated with siControl (siCt), two siRNAs targeting OCT4 (#1, #2) or NRF1 (#1, #2). (d) Microarray analysis was performed in DU145-CR and PC3-CR cells transfected with siControl, siOCT4 #1, or siNRF1 #1 (10 nM) for 72 h. Expression levels of OCT4 target genes (repressed (Fold < 0.5) or induced by siOCT4 (Fold > 2)) relative to siControl are shown by heatmaps. (e) Genes repressed by ribavirin treatment were enriched among those downregulated by siOCT4 among OCT4-binding genes (362 genes) in DU145-CR cells. Two-sided chi-square test was performed. Source data are provided as a Source Data file.

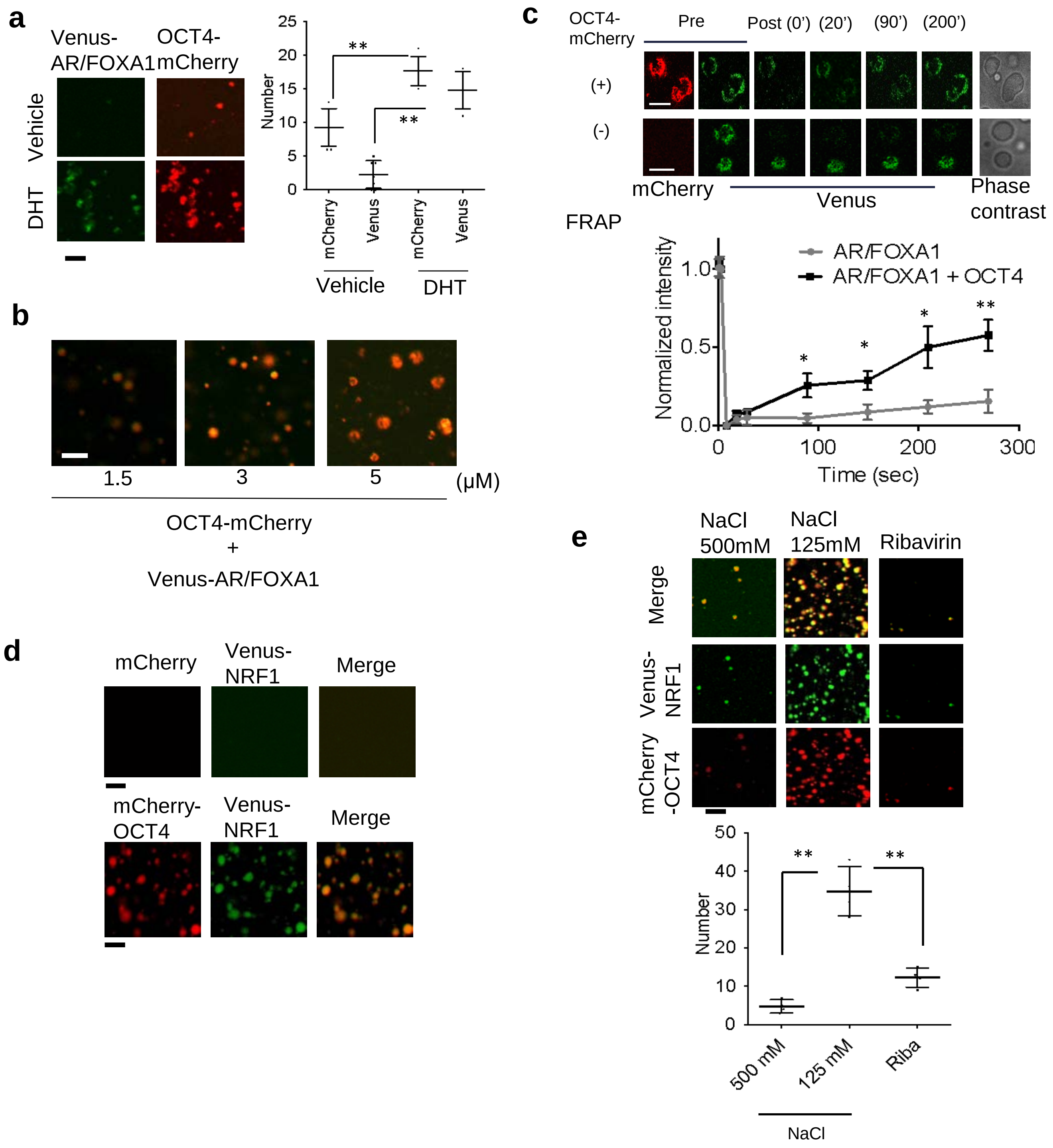

Supplementary Figure 9. Liquid droplet formation by collaborating TF complexes dependent on OCT4 in vitro. (a) Representative confocal images of droplet formation (total 10 μM) showing that DHT enhances droplet formation (Vehicle: N=7, DHT: N=6, biological independent images). **P < 0.01. Two-sided t-test was performed. Data are shown as average ± S.D. (b) Droplet formation dependent on the amount of added OCT4-mCherry. Droplet formation of mCherry-OCT4, Venus-AR (5 μM), and Venus-FOXA1 (5 μM), as indicated in droplet formation buffer with 125/500 mM NaCl and 10% PEG-8000. (c) FRAP kinetic plots and representative images from pre- and post-bleaching droplets formed with or without mCherry-OCT4 (N = 5, biological independent views). *P < 0.05, **P < 0.01. Two-sided t-test was performed. Data are shown as average ± S.D. (d) Representative images of droplet formation of mCherry, Venus, mCherry-OCT4, and Venus-NRF1 (total 10 μM), as indicated in droplet formation buffer with 125/500 mM NaCl and 10% PEG-8000. (e) Effects of high concentration of NaCl (500 mM) and ribavirin (Riba) (N=4, biological independent images). **P < 0.01. Two-sided t-test was performed. Data are shown as average ± S.D. Bar = 10 μm. Source data are provided as a Source Data file.

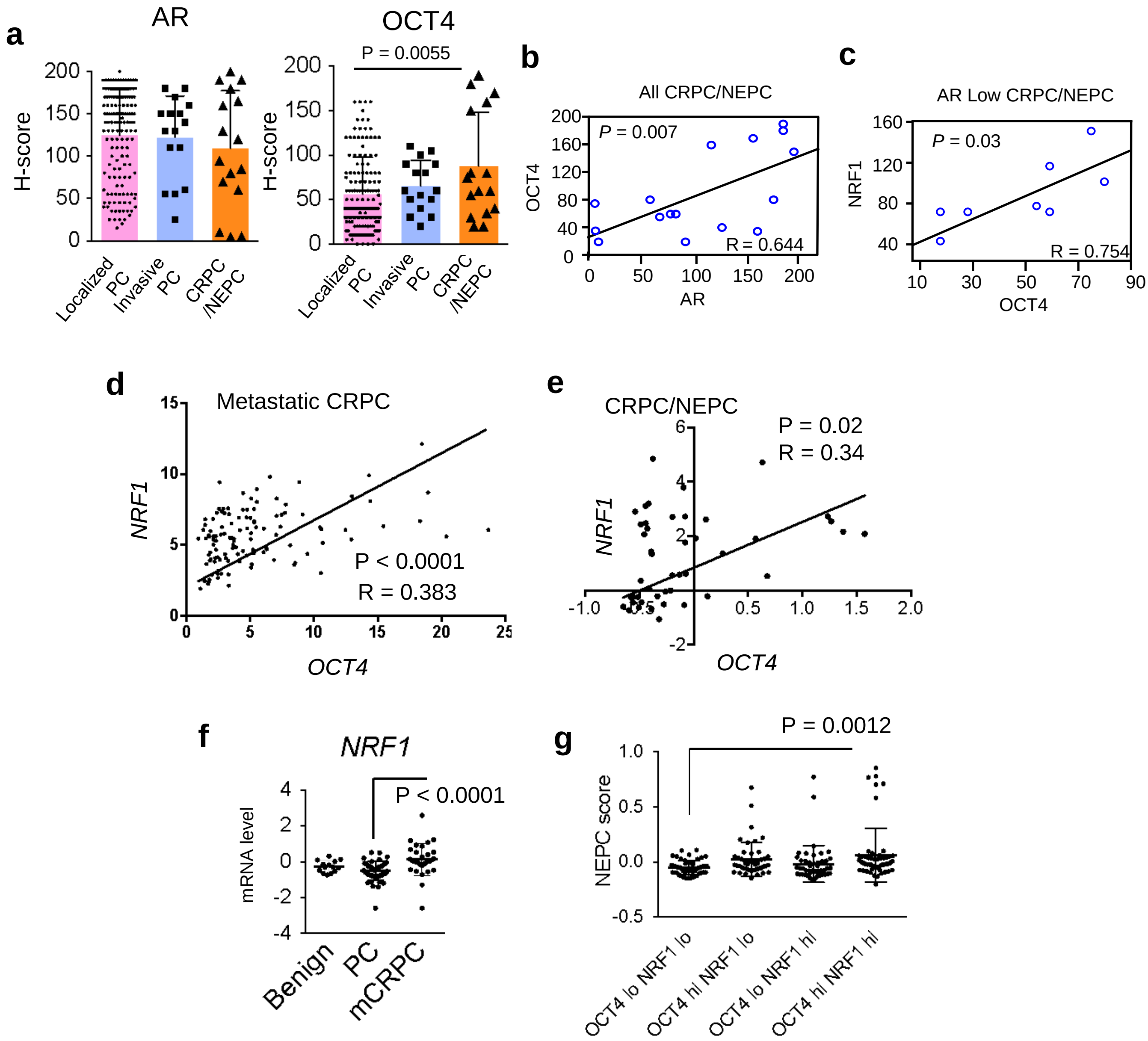

Supplementary Figure 10. Additional data of clinical analysis of OCT4 complex in PC. (a) OCT4 is upregulated in CRPC/NEPC tissues compared with localized PC. IHC scores are used to show the expression at the protein level. AR and OCT4 expression level in CRPC/NEPC tissues (N = 16) was compared with that of localized PC tissues (N = 143) or locally invasive PC (N = 16). One-way ANOVA and followed Bonferroni analysis was performed. Data are shown as average ± S.D. (b) High expression level of AR is associated with high expression of OCT4 in CRPC/NEPC tissues (N = 16). Regression analysis was performed to analyze the correlation. (c) OCT4 expression correlated with NRF1 in CRPC/NEPC tissues with low AR expression (N = 8). Regression analysis was performed to analyze the correlation. (d) Positive correlation between OCT4 and NRF1 in CRPC tissue samples (N = 118). RNA-seq (Robinson et al. 2015[53]) data was downloaded from cbioportal. Regression analysis was performed to analyze the correlation. (e) The expression level of OCT4 mRNA correlated with NRF1 in CRPC/NEPC tissues. RNA-seq data (Beltran et al., 2016[52]) was downloaded from cbioportal. Regression analysis was performed to analyze the correlation. (f) Expression level of NRF1 mRNA in benign prostate (N = 14), localized prostate cancer (PC) (N = 51), and metastatic CRPC (mCRPC) samples (N = 29). Publicly available independent data (GSE35988) was used to analyze the expression. Two-sided Mann-Whitney U-test was performed to determine P-value. Data are shown as average ± S.D. (g) High expression of OCT4 and NRF1 is associated with NEPC development. PC samples (N = 209) were classified to OCT4 high/low or NRF1 high/low groups defined by the median value. NEPC score (Abida et al. 2019[54]) was calculated using polyA RNA-seq data as described. Data was downloaded from cbioportal. One-way ANOVA and followed Dunnett’s test were used to compare groups. hi:high, lo:low. Data are shown as average ± S.D. Source data are provided as a Source Data file.

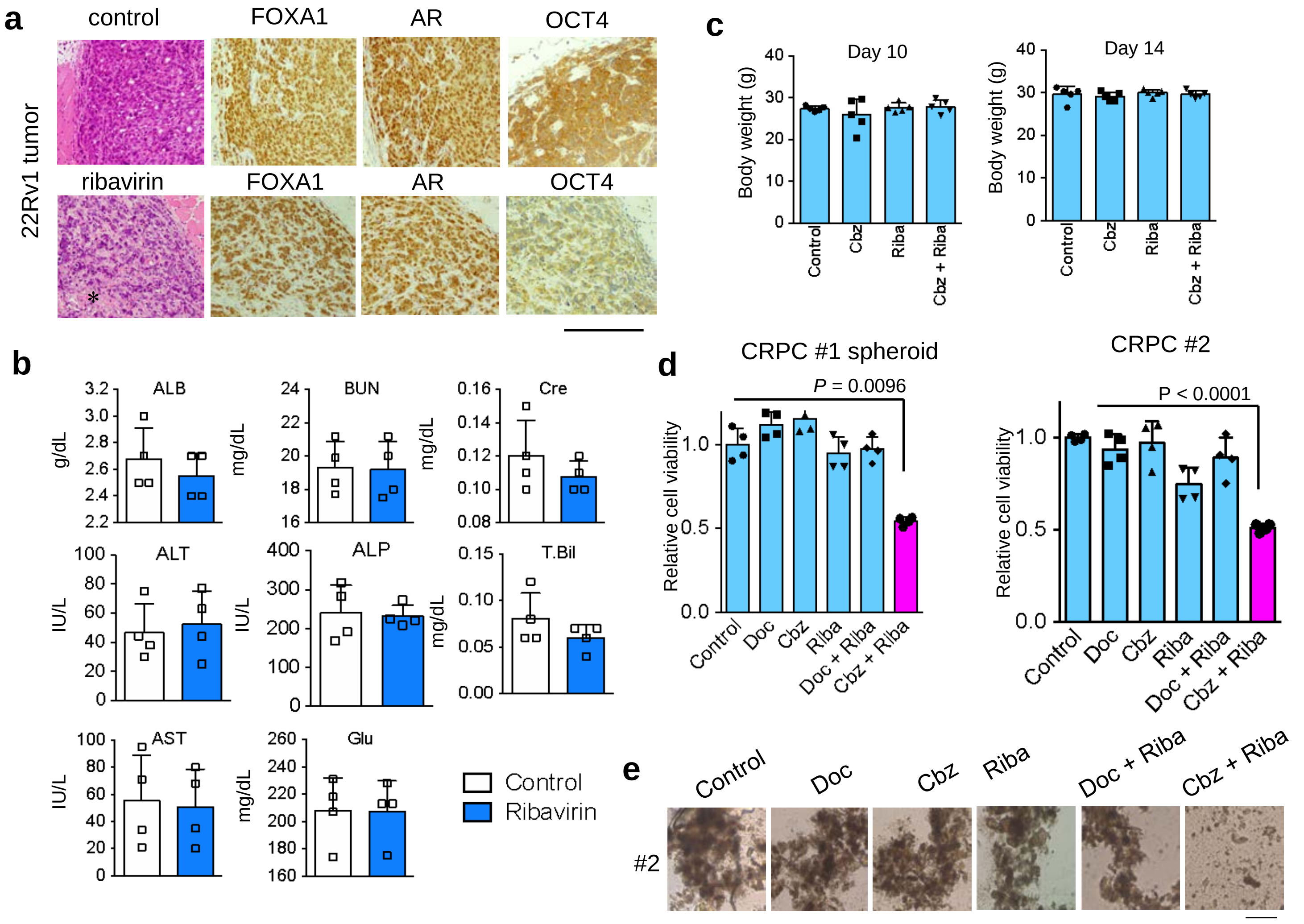

Supplementary Figure 11. Targeting OCT4-mediated complex is a potent strategy for the treatment of lethal PC cells. (a) IHC analysis of 22Rv1 tumors treated with vehicle or ribavirin. HE staining showed increased bionecrotic area (*) a by ribavirin treatment (left lower panel). AR, FOXA1 and OCT4 expression levels were also analyzed in these specimens by IHC. Protein level of OCT4 was markedly decreased by ribavirin (right lower panel), while FOXA1 and AR immunoreactivities were similar between these two groups. Bar = 100 µm. (b, c) No apparent toxic effect was observed in mice treated with ribavirin. Biochemical serum tests (b) and body weight measurements (c) were performed to analyze the effect of ribavirin treatment on several organs in mice. (b) Control: N=5, Ribavirin: N = 4, biological independent samples. Two-sided t-test was performed. (c) N=5, biological independent samples. One-way ANOVA was used to compare values. Data are shown as average ± S.D. (d) Spheroid formation was inhibited by addition of ribavirin. 100 µM ribavirin (Riba), 100 nM docetaxel (Doc), 100 nM cabazitaxel (Cbz) or vehicle was added to the medium of CRPC spheroids. Cell viability in patient-derived spheroids (CRPC #1, #2) was measured by luminometer using a CellTiter-Glo 3D cell viability assay kit (N=4, biological independent samples). Chemiluminescence values were normalized with the vehicle-treated samples. One-way ANOVA and followed Dunnett's test were used to compare groups. Data are shown as average ± S.D. (e) Representative images of patient-derived spheroids (CRPC #2) were shown. Bar = 10 µm. Source data are provided as a Source Data file.
