# Supplementary material for: Subtype-specific collaborative transcription factor networks are promoted by OCT4 in the progression of prostate cancer
Source: Nat Commun. 2021 Jun 18;12:3766. doi: 10.1038/s41467-021-23974-4 (PMC8213733; doi:10.1038/s41467-021-23974-4)
Supplement: Supplementary file 2 — Reporting Summary [file 41467_2021_23974_MOESM2_ESM.pdf]

nature research

Corresponding author(s): Satoshi Inoue

Last updated by author(s): May 6, 2021

Reporting Summary

Nature Research wishes to improve the reproducibility of the work that we publish. This form provides structure for consistency and transparency in reporting. For further information on Nature Research policies, see Authors & Referees and the Editorial Policy Checklist.

Statistics

For all statistical analyses, confirm that the following items are present in the figure legend, table legend, main text, or Methods section.

n/a	Confirmed	
☐	☒	The exact sample size (*n*) for each experimental group/condition, given as a discrete number and unit of measurement
☐	☒	A statement on whether measurements were taken from distinct samples or whether the same sample was measured repeatedly
☐	☒	The statistical test(s) used AND whether they are one- or two-sided<br>*Only common tests should be described solely by name; describe more complex techniques in the Methods section.*
☒	☐	A description of all covariates tested
☒	☐	A description of any assumptions or corrections, such as tests of normality and adjustment for multiple comparisons
☐	☒	A full description of the statistical parameters including central tendency (e.g. means) or other basic estimates (e.g. regression coefficient) AND variation (e.g. standard deviation) or associated estimates of uncertainty (e.g. confidence intervals)
☐	☒	For null hypothesis testing, the test statistic (e.g. *F*, *t*, *r*) with confidence intervals, effect sizes, degrees of freedom and *P* value noted<br>*Give P values as exact values whenever suitable.*
☒	☐	For Bayesian analysis, information on the choice of priors and Markov chain Monte Carlo settings
☒	☐	For hierarchical and complex designs, identification of the appropriate level for tests and full reporting of outcomes
☒	☐	Estimates of effect sizes (e.g. Cohen's *d*, Pearson's *r*), indicating how they were calculated

Our web collection on statistics for biologists contains articles on many of the points above.

Software and code

Policy information about availability of computer code

Data collection	MACS v1.4.2 and Bowtie 2 v. 2.2.6 were used for ChIP-seq. ROSE (http://younglab.wi.mit.edu/super_enhancer_code.html) was used for determining superenhancer regions. TopHat (v2.1.0) was used for RNA-seq analysis.
Data analysis	DAVID 6.7 (Pathway analysis), GraphPad Prism ver. 6.0 (chi-square test, Spearman correlation test, two way ANOVA), microsoft Excel v16.16.27 (two sided t-test), NGSPLOT (v 2.4.7.1)

For manuscripts utilizing custom algorithms or software that are central to the research but not yet described in published literature, software must be made available to editors/reviewers. We strongly encourage code deposition in a community repository (e.g. GitHub). See the Nature Research guidelines for submitting code & software for further information.

Data

Policy information about availability of data

All manuscripts must include a data availability statement. This statement should provide the following information, where applicable:

- Accession codes, unique identifiers, or web links for publicly available datasets
- A list of figures that have associated raw data
- A description of any restrictions on data availability

RNA-seq data have been deposited in the Japanese Genotype-phenotype Archive (JGA) under accession code JGAS00000000198 (https://humandbs.biosciencedbc.jp/en/hum0199-v1).
The Gene expression omnibus (GEO) accession numbers for sequence data (RNA-seq and ChIP-seq) used in this study are GSE141806 (https://www.ncbi.nlm.nih.gov/geo/query/acc.cgi?acc=GSE141806), GSE146656 (https://www.ncbi.nlm.nih.gov/geo/query/acc.cgi?acc=GSE146656), GSE146886 (https://www.ncbi.nlm.nih.gov/geo/query/acc.cgi?acc=GSE146886), and GSE123565 (https://www.ncbi.nlm.nih.gov/geo/query/acc.cgi?acc=GSE123565).
Publicly available data downloaded from GEO was GSE35988 (https://www.ncbi.nlm.nih.gov/geo/query/acc.cgi?acc=GSE35988). We used data of RNA-seq data (Beltran et al. Multi-Institute, Nat Med 2016, Robinson et al. SU2C/PCF Dream Team, Cell 2015, and Abida et al. SU2C/PCF Dream Team, PNAS 2019) in cbioportal (http://www.cbioportal.org/).

Field-specific reporting

Please select the one below that is the best fit for your research. If you are not sure, read the appropriate sections before making your selection.

☒ Life sciences ☐ Behavioural & social sciences ☐ Ecological, evolutionary & environmental sciences

For a reference copy of the document with all sections, see nature.com/documents/nr-reporting-summary-flat.pdf

Life sciences study design

All studies must disclose on these points even when the disclosure is negative.

Sample size	The sample size was determined based on the minimum requirement to perform statistical tests and the availability of materials. Studies done with cell lines were performed at least three times or using three samples. For studies using mice, sample size was at least four as indicated in the figure legends. The sample size xenograft studies is determined so as to be comparable in size to other similar experiments (such as Nat Commun. 2015 Sep 25;6:8219. doi: 10.1038/ncomms9219. or Nature . 2012 Jul 12;487(7406):239-43. doi: 10.1038/nature11125.), which has previously been demonstrated to be sufficient for determining the difference statistically.
Data exclusions	No data was excluded in these studies.
Replication	All experiments were reproduced at least twice. All attempts to replicate the experiments performed were successful.
Randomization	In the experiment of xenograft, the mice were randomly divided into two or four groups. For in vitro studies, cells cultured were allocated for the treatments randomly.
Blinding	No blind experiments was used in this study because the same investigator designed and conducted the experiments by setting proper controls.

Reporting for specific materials, systems and methods

We require information from authors about some types of materials, experimental systems and methods used in many studies. Here, indicate whether each material, system or method listed is relevant to your study. If you are not sure if a list item applies to your research, read the appropriate section before selecting a response.

Materials & experimental systems

n/a	Involved in the study	
☐	☒	Antibodies
☐	☒	Eukaryotic cell lines
☒	☐	Palaeontology
☐	☒	Animals and other organisms
☐	☒	Human research participants
☒	☐	Clinical data

Methods

n/a	Involved in the study	
☐	☒	ChIP-seq
☒	☐	Flow cytometry
☒	☐	MRI-based neuroimaging

Antibodies

Antibodies used	Rabbit polyclonal anti-HA (Y-11; 1:200 dilution), rabbit polyclonal anti-AR (H-280; 1:2000 dilution), mouse monoclonal anti-AR (sc-7305; 1:200 dilution, clone 441), and goat polyclonal anti-GAPDH (V-18; 1:1000 dilution) were purchased from Santa Cruz Biotechnology (Dallas, TX). Rabbit polyclonal anti-FOXA1 (ab23738; 1:2000 dilution), rabbit polyclonal anti-AR-V7 (ab198394; 1:200 dilution), rabbit polyclonal anti-OCT4 (ab181557/ab19857; 1:200 dilution), rabbit polyclonal anti-AcH3K27 (ab177178), rabbit polyclonal anti-K4me1 (ab8895), and mouse monoclonal anti-NRF1 (ab55744; 1:500 dilution, clone 2F9) were purchased from Abcam (Cambridge, UK). Mouse monoclonal anti-β-actin (A5441; 1:1000 dilution, clone AC-15) was purchased from Sigma. Mouse monoclonal anti-Flag (012-22384; 1:2000 dilution, clone 1E6) was purchased from Wako (Tokyo, Japan). Rabbit polyclonal anti-FOXM1(C15410232; 1:500 dilution) was purchased from diagenode (Liège, Belgium). Rabbit polyclonal anti-K4me3 (07-473) and rabbit polyclonal anti-RNA polII (05-623) were purchased from Millipore (Burlington, MA). Rabbit polyclonal anti-NRF2 (61600; 1:500 dilution) was purchased from Active Motif (Carlsbad, CA). Mouse monoclonal anti-TRIM25 (610570; 1:500 dilution, clone 2/EFP) was purchased from BD Biosciences (San. Diego, CA). Secondary antibodies were purchased from Amersham (Little Chalfont, UK, NA931, and NA934: Western blot analysis; 1:5000 dilution), BIO-RAD (Hercules, CA, STAR209P: Western blot analysis following immunoprecipitation; 1:300 dilution) and Jackson ImmunoResearch (West Grove, PA, 115-035-174: Western blot analysis following immunoprecipitation; 1:10000 dilution).
Validation	All antibodies for ChIP, IP, and western blot analysis were validated in our laboratory. Aplication of OCT4, FOXM1, FOXA1, K4me3, AcH3K27, NRF2, RNA polII for ChIP were also validated by the companies (Abcam, Millipore, Active motif).

Eukaryotic cell lines

Policy information about cell lines

Cell line source(s)	The cell lines used in the present study were obtained from ATCC. 22Rv1(ATCC CRL-250), PC3 (ATCC CRL-1435), LNCaP (ATCC CRL-1740), DU145 (ATCC HTB-81), VCaP (ATCC CRL-2876), and 293T (ATCC CRL-3216).
Authentication	Identities of the cells were confirmed by short tandem repeat (STR) analyses in 2019 and 2015 (BEX co. Ltd., Tokyo, Japan).
Mycoplasma contamination	We routinely checked for Mycoplasma contamination using a PCR-based kit, Mycoplasma detection kit (Jena Bioscience, Jena, Germany). Mycoplasma contaminated cells were discarded and not used for the experiments.
Commonly misidentified lines (See ICLAC register)	none

Animals and other organisms

Policy information about studies involving animals; ARRIVE guidelines recommended for reporting animal research

Laboratory animals	5-week-old male BALB/c nude mice (CLEA Japan) were used and housed in Animal Center at Tokyo Metropolitan Institute of Gerontology under standard condition of the room temperature range between 21 and 23 °C, the humidity of 50-60 % and semi natural light cycle of 12:12 hours light:dark.
Wild animals	None
Field-collected samples	None
Ethics oversight	Animal care was in accordance with the Tokyo Metropolitan Institute of Gerontology animal experiment guidelines. The ethics committee of animal experiments at the Tokyo Metropolitan Institute of Gerontology approved our study protocol.

Note that full information on the approval of the study protocol must also be provided in the manuscript.

Human research participants

Policy information about studies involving human research participants

Population characteristics	Human formalin-fixed paraffin-embedded primary (N = 159) and CRPC/NEPC (N = 16) tissues were collected from Keio University Hospital. The age of prostate cancer patients (all patients were male) ranged from 47-76 years and the pretreatment serum PSA levels were 0.5-29.54 ng/mL.
Recruitment	We obtained prostate cancer samples from surgeries, biopsies, and pathological anatomies. Written informed consent was obtained from each patient before treatment. No patients were recruited specifically for this study to avoid any biases.
Ethics oversight	The study was approved by the Human Genome, Gene Analysis Research Ethics Committee of the Tokyo Metropolitan Institute of Gerontology (#28-5961), and Keio University (#2016-0084).

Note that full information on the approval of the study protocol must also be provided in the manuscript.

ChIP-seq

Data deposition

☒ Confirm that both raw and final processed data have been deposited in a public database such as GEO.

☒ Confirm that you have deposited or provided access to graph files (e.g. BED files) for the called peaks.

Data access links *May remain private before publication.*	The Gene expression omnibus (GEO) accession numbers for sequence data used in this study are GSE146886 (https://www.ncbi.nlm.nih.gov/geo/query/acc.cgi?acc=GSE146886), and GSE123565 (https://www.ncbi.nlm.nih.gov/geo/query/acc.cgi?acc=GSE123565).
Files in database submission	ChIP-seq data (fastq and bed files) of FOXA1, AR, K4me1, AcH3 in GSE123565, ChIP-seq data (fastq and bed files) of OCT4, K4me3, AcH3K27, polII, FOXM1 and NRF1 in GSE146886.
Genome browser session (e.g. UCSC)	https://genome.ucsc.edu/s/Ken%2Dichi%20Takayama/hg19_prosate_2020_2

Methodology

Replicates	Duplicate experiments were performed for OCT4 ChIP-seq. Others were performed for one sample.
Sequencing depth	About ten million reads for each sample. Sequence tag was 50 bp and single end.

Antibodies	Rabbit polyclonal anti-AR Santa Cruz Biotechnology H-280, Rabbit polyclonal anti-FOXA1 Abcam ab23738, Rabbit polyclonal anti-OCT4 Abcam ab19857, Rabbit polyclonal anti-FOXM1 diagenode C15410232, Rabbit polyclonal anti-AcH3K27 Abcam ab177178, Rabbit polyclonal anti-K4me3 Millipore 07-473, Rabbit polyclonal anti-RNA polII Millipore 05-623, Mouse monoclonal anti-NRF1 Abcam ab55744
Peak calling parameters	Model-based analysis of ChIP-seq (MACS) was used for peak calling and the threshold for the binding sites was set as P < 1.0 E-4
Data quality	All obtained binding sites were P < 1e-4. OCT4 ChIP-seq: DHT) Fold > 3.25, FDR < 0.01, Vehicle) Fold > 2.51, FDR: 9980 among 13954 were FDR < 0.05, AR ChIP-seq: 48140 sites were > 5-fold, FDR: 47928 sites among 53809 were FDR < 0.05, FOXM1: FDR<1, Fold >2.23, Pol2: FDR<1, Fold > 2.86, 22Rv1 AcH3K27: Fold > 2.49, FDR < 5.34, DU145 OCT4 1) Fold > 4.24, 3425 among 3445 sites were > 5-fold, 2) 5399 among 5452 sites were > 5-fold, DU145_NRF1: Fold > 3.98, 4991 among 4999 sites were > 5-fold, NRF1 riba: Fold > 4.97, FDR < 1, AcH3K27_DU145 Fold > 3.8, FDR <5, Pol2_DU145 Fold 37882 among 46227 sites were > 5-fold.
Software	We used Trimmomatic-033 to trim and crop of Illumina (fastq) data by removing the sequences of adapters.<br>ChIP-seq reads were aligned to the hg19 genome assembly using Bowtie-1.1.2 with parameters -a --best --strata -m 1 -v 2.
